# Brain areas affected by intranasal oxytocin show higher oxytocin receptor expression

**DOI:** 10.1111/ejn.15447

**Published:** 2021-09-16

**Authors:** Philippe C. Habets, Christabel Mclain, Onno C. Meijer

**Affiliations:** ^1^ Division of Endocrinology, Department of Internal Medicine Leiden University Medical Center Leiden The Netherlands; ^2^ Department of Psychiatry, Department of Anatomy and Neurosciences Amsterdam University Medical Centre Amsterdam The Netherlands

**Keywords:** brain, fMRI, intranasal‐oxytocin, oxytocin, transcriptomics

## Abstract

Neuroimaging studies suggest that intranasal oxytocin (IN‐OXT) may modulate emotional and social processes by altering neural activity patterns. The extent of brain penetration after IN‐OXT is unclear, and it is currently unknown whether IN‐OXT can directly bind central oxytocin receptors (OXTRs). We investigated oxytocin pathway gene expression in regions affected by IN‐OXT on task‐based fMRI. We found that *OXTR* is more highly expressed in affected than unaffected subcortical regions; this effect did not vary by task type or sex. Cortical results revealed higher *OXTR* expression in regions affected by IN‐OXT in emotional processing tasks and in male‐only data. No significant differences were found in expression of the closely related vasopressin receptors. Our findings suggest that the mechanism by which IN‐OXT may alter brain functionality involves direct activation of central OXTRs.

## Graphical abstract

This work combines brain imaging data with brain transcriptomic atlas data. This work suggests that intranasal oxytocin (IN‐OXT) exerts its effects on affected brain areas by directly binding locally expressed OXTR in human. The local expression of OXTR in subcortical brain areas affected (on fMRI) by intranasal oxytocin (IN‐OXT) is significantly higher than in unaffected regions, independent of task type or gender.

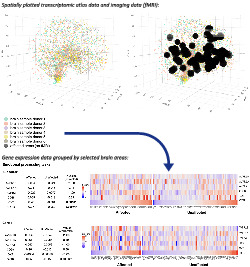

## 1 INTRODUCTION

Oxytocin is a neuropeptide, which has captured public and scientific interest in recent years due to its role in social behaviours and its potential as a novel treatment for neuropsychiatric disorders (Meyer‐Lindenberg et al., 2011). This interest has given rise to many neuroimaging studies investigating the effects of intranasal oxytocin (IN‐OXT) administration on brain activity (Grace et al., 2018). This body of research is contentious for two primary reasons: the neuroimaging results following IN‐OXT administration have been inconsistent (Grace et al., 2018; Quintana, 2018), and the mechanism of action by which IN‐OXT acts on the brain is unclear (Leng & Ludwig, 2016; Quintana et al., 2018). A recent study found that intranasal, but not intravenous, OXT administration in Macaques resulted in quantifiable exogenous OXT levels in multiple brain regions (Lee et al., 2020). This finding suggests that IN‐OXT may bypass the blood–brain barrier to act directly on the brain, as has been posited before (Erdő et al., 2018). However, penetrance was quite low and highly variable across animals in this study (Lee et al., 2020), casting additional doubt on the efficacy of IN‐OXT delivery.

A direct mechanism of action would imply the ability of IN‐OXT to bind to OXTRs in the brain. The distribution of receptors and binding patterns can be inferred from *OXTR* mRNA data. In addition to *OXTR* and *OXT* (the gene coding for the oxytocin prepropeptide), *CD38* is a crucial oxytocin pathway gene, as the CD38 protein mediates intracellular Ca^2+^ mobilization necessary for peptide release from soma and axon terminals of hypothalamic OXT neurons (Jin et al., 2007). Together, *OXTR*, *OXT* and *CD38* are key elements of the oxytocin signalling pathway that have been frequently implicated in human social behaviour (Heinrichs & Domes, 2008; Jin et al., 2007; Jurek & Neumann, 2018; Quintana et al., 2019). Expression of these three genes is intercorrelated and highly variable throughout the subcortical human brain, with specific subcortical structures showing enrichment for these genes (including the hypothalamus) (Quintana et al., 2019). Recent RNA sequencing datasets show low but variable expression of *OXTR* in multiple human cortical areas, while cortical expression of *OXT* and *CD38* in the same RNA‐seq datasets is hardly detectable with no measured variance (Allen Institute, 2019; Hodge et al., 2019).

**FIGURE 1:**
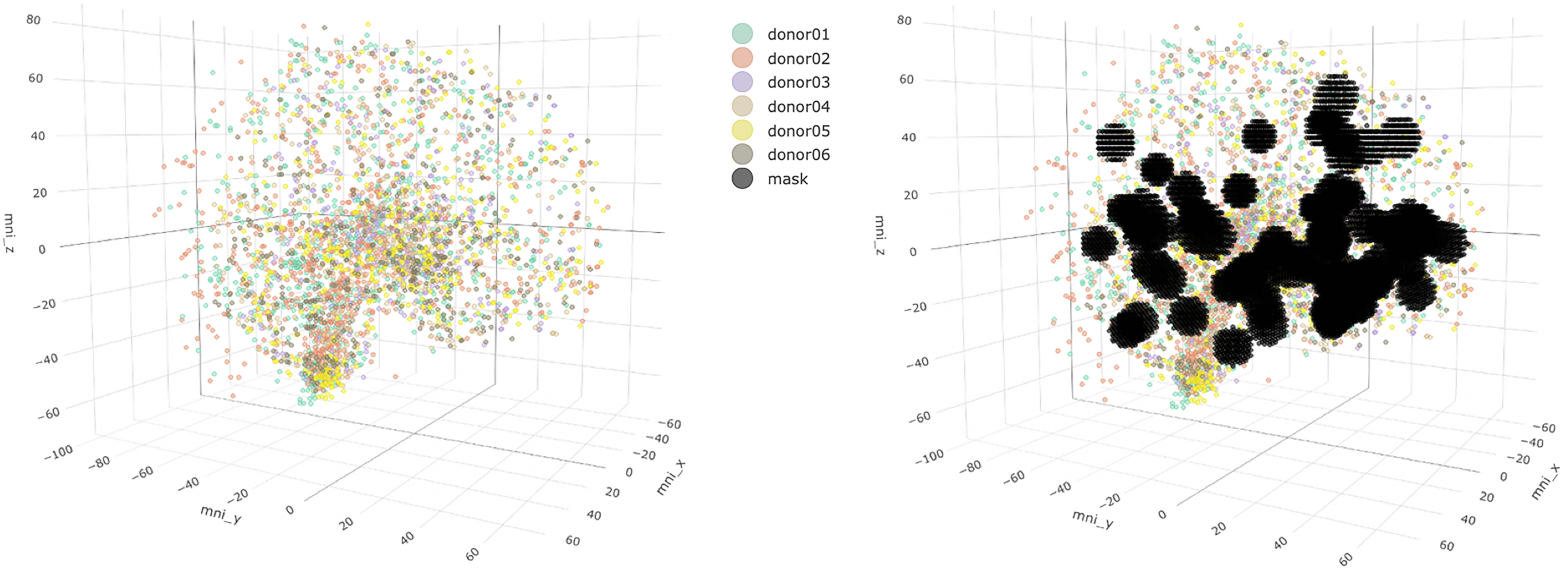
Data plotted in MNI‐152 space. Brain samples from all six donors plotted in MNI‐152 space (left). On the right, the thresholded *p*‐statistic map for emotional processing is plotted in the same space

Here, we investigate expression of *OXT*, *OXTR* and *CD38* in regions significantly affected by IN‐OXT on task‐based fMRI in humans. ‘Affected’ is defined as showing increased activity in IN‐OXT over placebo conditions. We combined the spatial expression patterns from microarray data from the Allen Human Brain Atlas (AHBA) (Hawrylycz et al., 2012) with fMRI metadata from 39 fMRI studies included in a recent meta‐analysis study (Grace et al., 2018). Technically, we spatially map AHBA samples to fMRI brain data in order to relate local gene expression of the three genes to effects observed in the collated fMRI data (see Section 2). Figure 1 shows a visual representation of overlapping AHBA samples with IN‐OXT ‘affected’ areas from the fMRI data. We also investigate vasopressin receptor (*AVPR*) expression to control for possible IN‐OXT‐mediated fMRI effects via AVPR binding, as AVPRs have (weak) affinity for OXT and play an interrelated role in regulating social cognition and behaviour (Heinrichs & Domes, 2008; Meyer‐Lindenberg et al., 2011). Earlier work reported spatially correlated expression of *OXTR* and *AVPR* (Quintana et al., 2019). We further investigate whether spatial patterns of *OXTR* co‐expression, with respect to both *AVPR* and *OXT/CD38*, are different for IN‐OXT affected and unaffected brain regions. Accordingly, we assess spatial expression correlations between all genes of interest (*OXT*, *OXTR*, *CD38*, *AVPR1A*, *AVPR1B* and *AVPR2*) to determine whether spatial co‐expression patterns differ in regions affected by IN‐OXT on fMRI. The statistic maps of the earlier collated 39 imaging studies were categorized into tasks of emotion perception or processing, and those that probed other social cognitive processes (Grace et al., 2018). Using a stringent activation likelihood estimation (ALE) method, Grace et al. identified a cluster of convergence in experiments probing emotional, but not social, processes (Grace et al., 2018). Given the inconsistency in fMRI foci activated by IN‐OXT and the poor penetrance via nasal delivery shown in animal models (Lee et al., 2020), we hypothesized that brain regions affected by IN‐OXT on task‐based fMRI would not show significantly higher expression of OXTR in comparison with unaffected brain regions.

## 2 MATERIAL AND METHODS

All code and used files are made publicly available on GitHub (https://github.com/pchabets/fMRI-Transcriptomics-Oxytocin).

### 2.1 Transcriptomic atlas

The Allen Human Brain Atlas (AHBA) is a publicly available transcriptional atlas based on microarray measures in 3702 samples across brainstem, cerebellum, subcortical and cortical brain structures across six postmortem human brains (five male and one female) (Hawrylycz et al., 2012). For limited samples of two donor brains, expression values were also measured by RNA sequencing. All expression data and metadata were downloaded from the AHBA (http://human.brain-map.org) (Hawrylycz et al., 2012, 2015).

### 2.2 fMRI data

Data from 39 selected IN‐OXT fMRI studies were included in the study by Grace et al. (2018). For our analysis, we looked at four different *p*‐statistic maps. Each statistic map was created separately for differential activation in the intranasal‐oxytocin versus placebo conditions (OXT > PBO) in the following:

1. tasks related to emotional processing (14 experiments, 72 foci, 506 participants)
2. tasks related to social processing (16 experiments, 153 foci, 873 participants)
3. all experiments that looked at the OXT > PBO contrast (31 experiments, 243 foci, 1420 participants)
4. all experiments that looked at the OXT > PBO contrast but including data from male participants only (22 experiments, 142 foci, 855 participants)

Of the 39 included studies, 23 used within‐subject comparisons, while 16 used between‐subject comparisons. Between‐subject studies had various justifications for using this methodology, such as the potentially confounding effects of repetitive fear conditioning in experiments using this paradigm (Eckstein et al., 2016). Oxytocin dosage was similar for the vast majority of studies: a dosage of 24 International Units (IU) was used for 33 included studies, where 26 IU, 32 IU and 40 IU were used in one, two and three studies, respectively. Descriptive data on all included 39 studies are listed in Table S2. Further details about study selection, data processing, ALE analysis and statistic map generation is described in the original paper by Grace et al. (2018).

All four *p*‐statistic maps were downloaded from Neurovault at (https://neurovault.org/collections/3713/) (map ID: 63321, 63369, 63357, 63349). Each statistic map was then thresholded for a *p*‐value of 0.05. The resulting thresholded foci were considered as ‘IN‐OXT‐affected brain areas’, and assessed for differential gene expression in comparison to other brain areas that, after thresholding, were considered ‘IN‐OXT‐unaffected’ (Figure 1). As OXTR activation is normally linked to activating a set of signalling cascades (Jurek & Neumann, 2018), we focused on brain areas showing higher activation after IN‐OXT only (OXT > PBO), not brain areas showing lower activation after IN‐OXT (PBO > OXT).

### 2.3 Data analysis

With the exception of probe re‐annotation, R was used for all data analysis, with all used packages installed under R version 4.0.

First, we re‐annotated all microarray probes for the genes of interest to the latest human genome version and reference sequence (20 May 2020), using the Re‐Annotator package (Arloth et al., 2015). Re‐Annotator is freely available for download online (https://sourceforge.net/projects/reannotator/).

Next, given that more than one probe was annotated to each gene of interest (*OXT*, *OXTR*, *CD38*, *AVPR1A*, *AVPR1B* and *AVPR2*), we selected a probe for each gene on the basis of highest expression (intensity analysis). We validated probe selections by confirming that each probe also showed the highest correlation (Spearman's ρ) with the RNA sequencing measures for the same gene, from the same brain sample (data only available for the first two donor brains) (Arnatkevic Iute et al., 2019).

AHBA samples and fMRI masks were plotted in MNI‐152 space. Using trilinear interpolation, we calculated for each AHBA sample whether it could be assigned to an affected brain area (meaning falling inside the fMRI mask) or not. Brainstem and cerebellum samples were excluded from analysis. Because the rate of ‘affected’ versus ‘unaffected’ samples differed between cortical and subcortical samples for all statistic maps (Table 1), we separated cortical and subcortical samples in our analyses to prevent bias by the inherently different gene expression profiles of the cortex and subcortex (Hawrylycz et al., 2015). This resulted in the selection of samples that were categorized in one of four groups:

1. subcortical IN‐OXT unaffected samples;
2. subcortical IN‐OXT affected samples;
3. cortical IN‐OXT unaffected samples;
4. cortical IN‐OXT affected samples.

**TABLE 1:** Included samples

		Emotion processing	Social processing	Male only	All tasks
Subcortical samples	Affected	177	193	176	208
	Unaffected	809	793	705	778
Cortical samples	Affected	147	103	111	138
	Unaffected	1615	1659	1427	1624

We corrected downloaded expression values from the AHBA for any between‐donor differences that might drive differential gene expression findings by using the *removeBatchEffect()* function from the limma package for R (Ritchie et al., 2015), treating each donor as a separate batch. It is relevant to note that gene expression patterning across brain structures was assessed for reproducibility in all six AHBA donor brains in previous works (Hawrylycz et al., 2015; Quintana et al., 2019). This was done using the differential stability (DS) metric: a measure for the consistency of a gene's differential expression pattern across brain structures (Shaw et al., 2011). In one study that included 17,348 protein coding genes in the DS analysis, all oxytocin pathway genes showed a DS of 0.65 or higher, with *OXTR* and *OXT* in the top 10% of genes with highest DS scores (see table S2 of Hawrylycz et al., 2015). Another study by Quintana et al. found *OXTR* and *CD38* to both have top decile DS scores in a list of 20,737 protein coding genes (Quintana et al., 2019). These results suggests that, at least in the six donor brains, oxytocin pathway genes show gene expression patterning that is reproducible across donor brains, regardless of individual differences like sex or age.

Next, we *z*‐normalized expression values across brains for use in heatmap visualization and further assessment. Data from all six donor brains were used for assessing differential gene expression in affected versus unaffected brain areas in the first three *p*‐statistic maps. For the *p*‐statistic map that includes male participants only, expression data from only the five male donor brains were used.

We assessed differential gene expression between affected and unaffected samples separately for subcortical and cortical samples using a Wilcoxon‐rank sum test. We controlled for multiple testing by using Bonferroni correction of *p*‐values. For all our analyses we used Bonferroni‐corrected *p* < 0.05 as an indication of significance. All steps after probe selection were repeated for the four different fMRI masks.

We assessed correlations between all genes of interest in IN‐OXT‐affected and IN‐OXT‐unaffected brain areas, using the *p*‐statistic map of all experiments that looked at the OXT > PBO contrast. We calculated Pearson's *r* for both groups of samples (subcortical affected vs. unaffected, cortical affected vs. unaffected respectively), incorporating expression data from all six donor brains. This resulted in four correlation matrices. Pairs of correlation matrices corresponding to affected and unaffected samples in the subcortical and cortical subgroups were respectively tested for equality, using the *cortest.normal()* function of the ‘psych’ package for R (this function uses the Steiger test for comparing correlation matrices) (Revelle, 2020).

After differences in overall gene correlation matrices had been assessed (only significant for subcortical groups, see Section 3), we tested for *OXTR* co‐expression differences (i.e., differences in Pearson's *r*) in the subcortical affected versus unaffected samples. We first excluded insignificant gene correlation coefficients (Bonferroni‐corrected *p* ≥ 0.05) in both affected versus unaffected samples. We assessed significance in differences between the remaining correlation coefficients for *OXTR* and the other genes of interest using Fisher's *Z*‐transformation test (two‐tailed, Bonferroni‐corrected *p* < 0.05).

## 3 RESULTS

### 3.1 IN‐OXT affected subcortical brain areas show higher expression of OXTR

**FIGURE 2:**
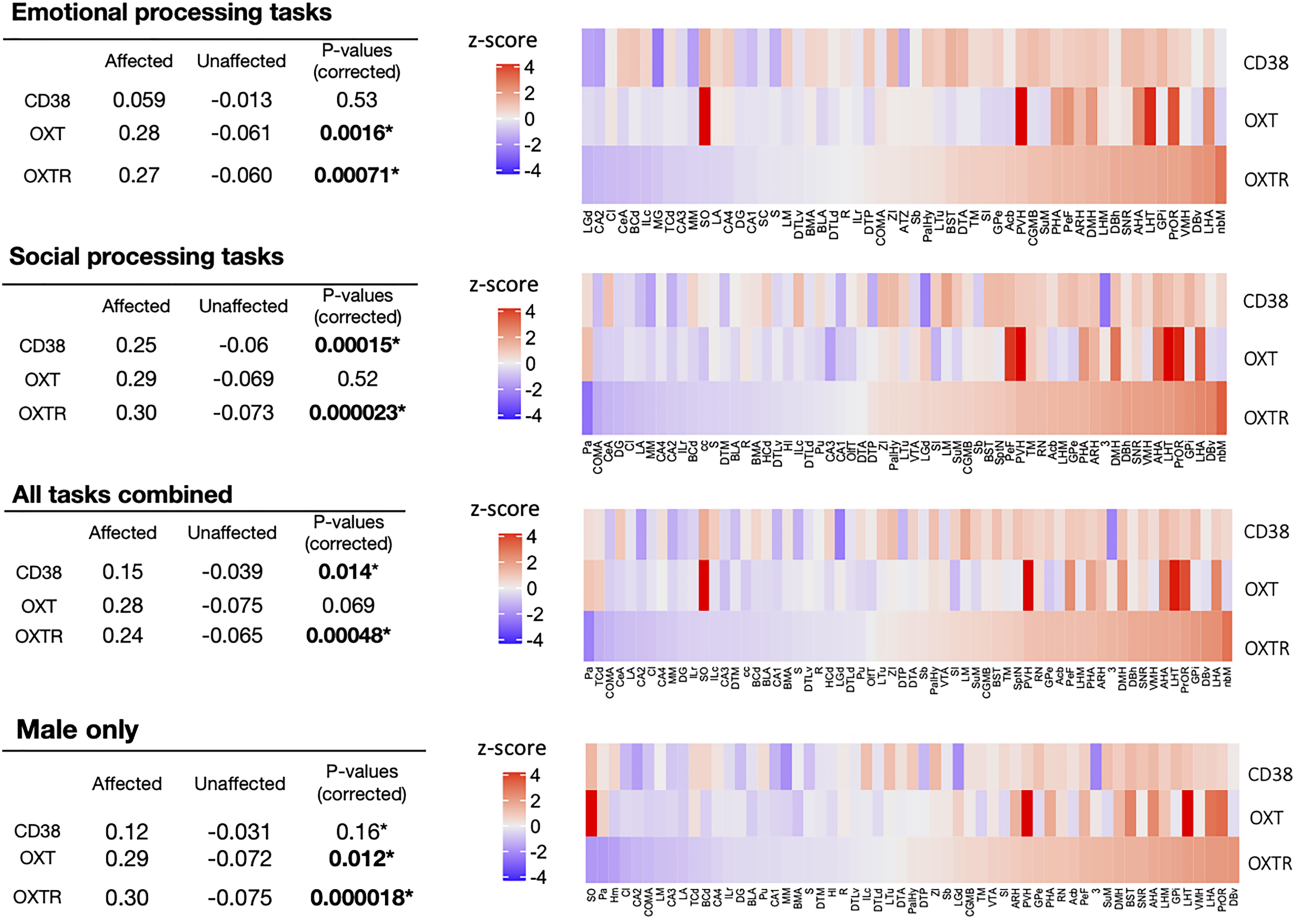
Differential expression results. Differences in average *z*‐normalized expression values for *CD38*, *OXT* and *OXTR* in affected versus unaffected subcortical samples using four different *p*‐statistic maps. Heatmaps show average expression of genes for brain structures that include affected samples. Brain structure abbreviations are adopted from the Allen Human Brain Atlas (AHBA) data

Expression data for IN‐OXT affected and unaffected subcortical samples in the emotion processing, social processing, combined, and male‐only masks are shown in Figure 2. For all four fMRI thresholded *p*‐statistic maps, samples from IN‐OXT affected subcortical brain regions showed significantly higher average expression of *OXTR* compared with unaffected subcortical brain regions. Oxytocin pathway genes *OXT* and *CD38* were found to show a similar but weaker effect compared with *OXTR*, not reaching significance in all settings. No significant difference in subcortical expression was found for any of the AVP receptors.

### 3.2 IN‐OXT affected cortical brain areas show differential expression for some OXT pathway genes

For cortical regions, areas affected by IN‐OXT in emotional processing tasks showed significantly higher expression of *OXTR* (corrected *p* < 0.001). The same result was found for affected cortical regions in the male‐only *p*‐statistic map across all tasks (corrected *p* < 0.05). However, no significant difference in cortical *OXTR* expression was found in the other *p*‐statistic maps (social processing and all combined OXT > PBO experiments). Except for the social processing *p*‐statistic map (corrected *p* < 0.05), *OXT* showed no cortical differential expression in any of the included fMRI masks. With the exception of the emotional processing *p*‐statistic map, a significantly higher expression of *CD38* in affected cortical brain areas was found in all other masks (corrected *p* < 0.05). There was no significant difference in cortical expression of the AVP receptors in any of the fMRI statistic maps.

### 3.3 IN‐OXT affected subcortical brain areas show higher OXTR‐AVPR1a co‐expression

**FIGURE 3:**
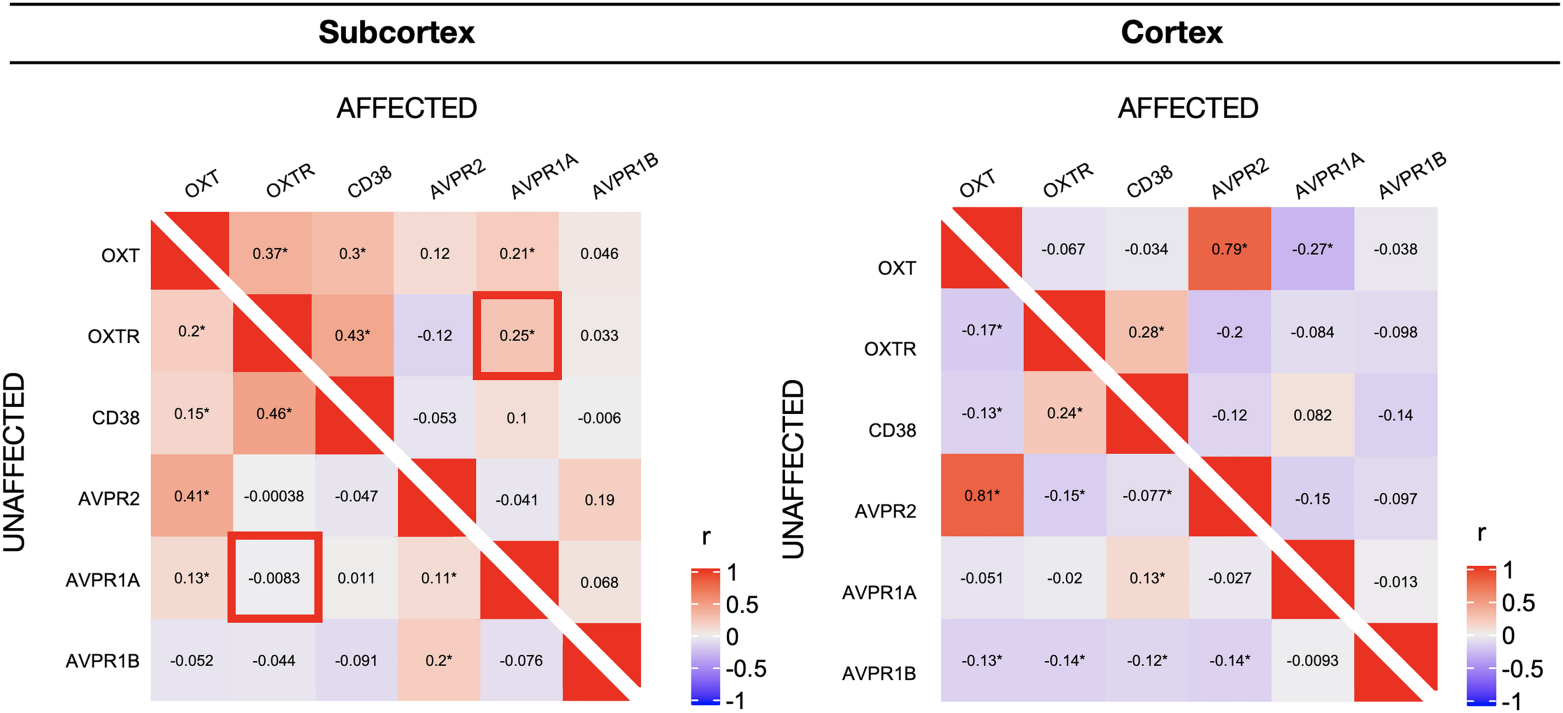
Correlations. Pairwise correlations (Pearson's *r*) of genes of interest in affected and unaffected samples, in subcortex and cortex respectively, using the all‐task *p*‐statistic map. Significant correlations (corrected *p* < 0.05) are marked with an asterisk (*). The significant difference in correlation of *OXTR*‐*AVPR1A* between affected versus unaffected subcortical areas is highlighted in the left panel

Pairwise gene correlations in affected samples (using the *p*‐statistic map that combines all fMRI OXT > PBO experiments) are shown in Figure 3 for subcortex and cortex separately. Correlation matrices of subcortical affected versus unaffected samples differed significantly (*χ* ^2^ = 53.55, *p* = 3.12e‐6). Correlation matrices for the cortical samples showed no significant difference between affected versus unaffected samples (*χ* ^2^ = 14.18, *p* = 0.51). Next, comparing *OXTR* co‐expression in subcortical affected versus unaffected samples, a significant difference was found only for the co‐expression of *OXTR* and *AVPR1a* (Figure 3)*. OXTR* and *AVPR1a* showed a significantly higher co‐expression in the affected samples (*r* = 0.253 vs. *r* = −0.00832, corrected *p* = 0.0026).

## 4 DISCUSSION

Contrary to our hypothesis, we have identified robust differences in oxytocin pathway gene expression between IN‐OXT‐affected versus IN‐OXT‐unaffected subcortical brain regions. Remarkably, *OXTR* is more highly expressed in affected subcortical regions across all assessed fMRI files. As blood–brain barrier penetration of IN‐OXT is poor (Lee et al., 2020; Quintana et al., 2018), our findings seem to support the hypothesis that IN‐OXT acts directly on the brain via binding to its receptors in at least subcortical affected brain areas. Notwithstanding, recent evidence shows IN‐OXT effects on some brain regions can be (partially) explained by rising systemic OXT concentration following intranasal administration (Martins et al., 2020). It is therefore possible that IN‐OXT indeed affects brain regions by binding the locally expressed OXTR directly, but this OXT can be (partially) delivered systemically following intranasal administration.

Cortical results were less consistent, with higher *OXTR* expression in affected areas on the emotion processing and male masks only. It is notable that the emotion processing file was the only mask for which the initial ALE meta‐analysis by Grace et al. revealed a significant cluster of convergence (Grace et al., 2018). Tasks probing emotion most commonly involved facial emotion recognition or discrimination, while ‘social tasks’ covered a much wider range (see table S3 of the report by Grace et al., 2018). This may have affected the specificity of the affected/unaffected regions on the social mask. Moreover, recent RNA‐sequencing data indicate that cortical expression of *OXTR* is low, while *OXT* and *CD38* is hardly detectable with no measured variance (Allen Institute, 2019). Subcortical results thus provide stronger evidence from which to draw our conclusions. Although the thresholding of *p*‐statistic maps is to some extent arbitrary, the risk of including false positive affected areas also comes with the risk of diluting any significant differential gene expression effect. It is therefore noteworthy that subcortical differential *OXTR* expression is found to be robust in all four fMRI masks.

The most consistent subcortical areas with high OXTR expression associated with neuronal activation included the nbM (basal nucleus of Meynert), DBv (nucleus of the diagonal band), PrOR (preoptic regions), GPi (*globus pallidus*) and LHA, VMH, LHT, AHA (hypothalamic areas). Some of these, like the nbM, have widespread projections to higher brain areas. The GPi has been implicated in social processes (Grace et al., 2018), and the nbM primarily consists of cholinergic neurons that are part of a system associated with several cognitive and behavioural functions (Lew & Semendeferi, 2017). Especially in the case of the nbM, where over 90% of the principal neurons are cholinergic, ACh may be one of the secondary mediators of IN‐OXT in cortical areas (Lew & Semendeferi, 2017). Receptor‐activity correlation results for all brain regions are graphically listed as heatmaps in Figures S1–S4. Cortical areas seem to be less consistent in their association of high *OXTR* expression and neuronal activation. One explanation for this could be that cortical activation processes are more specific to different tasks (e.g., emotional processing vs. social processing tasks). Importantly, the signal of the microarray probes that measure *OXTR* expression, although present in some parts, is rather weak in cortical areas compared to subcortical regions (Allen Institute, 2019). Any differential expression analysis based on weak probe signals might be more subjective to artefacts in the inherently noisy data of microarray probes. This should be taken into account when interpreting the cortical region‐specific results plotted in Figures S1–S4.

*OXTR* and *AVPR1a* show significantly higher co‐expression in affected versus unaffected subcortical brain areas. A higher correlation between these receptors does not necessarily indicate an interdependency between them (Galbusera et al., 2017). Rather, this co‐expression may signify brain areas that have a function in multiple behavioural processes, some of which rely on OXTR and some on AVPRs. Thus, while IN‐OXT mediated activity changes are specific to OXTR binding, affected brain areas likely play a role in other functions which may be sensitive to AVP.

The AHBA provides the most detailed dataset for examining spatial distribution of human brain transcriptomics to date but is limited to six donor brains. It is therefore important to note that independent sample validation of our genes of interest was performed in previous work using 10 overlapping brain regions from the Gentotype‐Tissue Expression (GTEx) project (Quintana et al., 2019). *OXTR* and *CD38*, but not *OXT*, showed a significant correlation in expression between both datasets (Quintana et al., 2019). Despite substantial differences in spatial coverage and donor number (for GTEx 88 to 173 donors are included depending on the brain region), brain co‐expression patterns of oxytocin pathway genes were found to be similar for the comparable 10 brain regions (details described in Quintana et al., 2019). This lends validity to our use of the AHBA for analysis.

## 5 CONCLUSION

We straightforwardly tested whether brain areas sensitive to OXT are enriched in OXT receptors (and associated factors). The answer is clearly confirmative for subcortical brain areas and much more nuanced for cortical areas. By providing evidence for a hypothesized mechanism of action, our findings serve to attenuate scepticism towards the ability of IN‐OXT to act directly on the brain and thereby affect brain functionality.

## CONFLICT OF INTEREST

The authors declare no conflict of interest.

## AUTHOR CONTRIBUTIONS

P. C. H. and O. C. M. designed research. P. C. H. and C. M. performed research. P. C. M. and C. M. analysed data. P. C. M., C. M. and O. C. M. wrote the paper.

## FUNDING

The authors received no financial support for the research, authorship and/or publication of this article.

### PEER REVIEW

The peer review history for this article is available at https://publons.com/publon/10.1111/ejn.15447.

## Supporting information


**Table S1.** List of all AHBA abbreviations for structure acronyms.

**Table S2.** Table of descriptive data of all 39 fMRI studies included in the meta‐analysis by Grace et al.

**Figure S1. Emotional processing results for all genes.** Differences in average z‐normalized expression values for all genes of interest in affected versus unaffected subcortical and cortical samples using the emotional processing p‐statistic map. Heatmaps show average expression of genes per brain structure. Brain structure abbreviations are adopted from the AHBA data.

**Figure S2. Social processing results for all genes.** Similar to S1 Fig, but results are shown for the social processing mask.

**Figure S3. All tasks results for all genes.** Similar to S1 Fig, but results are shown for the all‐tasks map.

**Figure S4. Male‐only results for all genes.** Similar to S1 Fig, but results are shown for the male‐only all‐tasks map, with only five male donor brains included in the analysis.

**Data S1**. Supplementary Files.

## Data Availability

All data and code associated with this paper is made available for publication and accessible online (https://github.com/pchabets/fMRI-Transcriptomics-Oxytocin).
