# Supplementary material for: Brain areas affected by intranasal oxytocin show higher oxytocin receptor expression
Source: Eur J Neurosci. 2021 Sep 16;54(7):6374–81. doi: 10.1111/ejn.15447 (PMC9291869; doi:10.1111/ejn.15447)
Supplement: Supplementary file 1 — Table S1. List of all AHBA abbreviations for structure acronyms. [file EJN-54-6374-s003.docx]

**Supplemental Data**

Structure acronyms (as they appear in Figure 2) and their corresponding names, extracted from the AHBA sample information for all six donor brains.

| **Stucture Acronym** | **Structure Name** |
| --- | --- |
| PCLa-i | paracentral lobule, anterior part, right, inferior bank of gyrus |
| Cl | claustrum, right |
| LGd | dorsal lateral geniculate nucleus, left |
| CA4 | CA4 field, right |
| DG | dentate gyrus, right |
| Dt | dentate nucleus, left |
| Fas | fastigial nucleus, left |
| Emb | emboliform nucleus, right |
| S | subiculum, left |
| CA1 | CA1 field, left |
| TCd | tail of caudate nucleus, left |
| DTA | anterior group of nuclei, right |
| DTLv | lateral group of nuclei, right, ventral division |
| ILr | rostral group of intralaminar nuclei, right |
| DTM | medial group of nuclei, left |
| ILc | caudal group of intralaminar nuclei, left |
| R | reticular nucleus of thalamus, right |
| Pa | paraventricular nuclei, right of thalamus, right |
| SI | substantia innominata, right |
| Sb | subthalamic nucleus, right |
| SNC | substantia nigra, pars compacta, left |
| ZI | zona incerta, left |
| CGMB | central gray substance of midbrain, right |
| RN | red nucleus, left |
| SNR | substantia nigra, pars reticulata, left |
| MTG-i | middle temporal gyrus, left, inferior bank of gyrus |
| ATZ | amygdalohippocampal transition zone, left |
| BLA | basolateral nucleus, left |
| BMA | basomedial nucleus, left |
| LA | lateral nucleus, left |
| CeA | central nucleus, left |
| SIG | short insular gyri, left |
| PoG-cs | postcentral gyrus, right, bank of the central sulcus |
| OTG-i | occipito-temporal gyrus, left, inferior bank of gyrus |
| FuG-its | fusiform gyrus, left, bank of the its |
| FuG-l | fusiform gyrus, left, lateral bank of gyrus |
| FuG-cos | fusiform gyrus, left, bank of cos |
| HG | Heschl's gyrus, left |
| Pu | putamen, left |
| LIG | long insular gyri, right |
| STG-i | superior temporal gyrus, left, inferior bank of gyrus |
| MTG-s | middle temporal gyrus, left, superior bank of gyrus |
| ITG-l | inferior temporal gyrus, left, lateral bank of gyrus |
| ITG-mts | inferior temporal gyrus, left, bank of mts |
| STG-l | superior temporal gyrus, right, lateral bank of gyrus |
| ITG-its | inferior temporal gyrus, right, bank of the its |
| PoG-il | postcentral gyrus, right, inferior lateral aspect of gyrus |
| PLT | planum temporale, right |
| PrG-prc | precentral gyrus, left, bank of the precentral sulcus |
| PrG-sl | precentral gyrus, left, superior lateral aspect of gyrus |
| PrG-il | precentral gyrus, left, inferior lateral aspect of gyrus |
| MFG-i | middle frontal gyrus, left, inferior bank of gyrus |
| SFG-m | superior frontal gyrus, left, medial bank of gyrus |
| SFG-l | superior frontal gyrus, left, lateral bank of gyrus |
| MFG-s | middle frontal gyrus, left, superior bank of gyrus |
| PrG-cs | precentral gyrus, right, bank of the central sulcus |
| GPi | globus pallidus, internal segment, right |
| PHG-l | parahippocampal gyrus, left, lateral bank of gyrus |
| PHG-cos | parahippocampal gyrus, left, bank of the cos |
| CgGp-s | cingulate gyrus, parietal part, left, superior bank of gyrus |
| CgGp-i | cingulate gyrus, parietal part, left, inferior bank of gyrus |
| cc | corpus callosum |
| cgb | cingulum bundle, right |
| PLP | planum polare, left |
| SMG-s | supramarginal gyrus, left, superior bank of gyrus |
| BCd | body of caudate nucleus, right |
| SMG-i | supramarginal gyrus, left, inferior bank of gyrus |
| CgGf-s | cingulate gyrus, frontal part, left, superior bank of gyrus |
| CgGf-i | cingulate gyrus, frontal part, left, inferior bank of gyrus |
| PoG-sl | postcentral gyrus, right, superior lateral aspect of gyrus |
| GPe | globus pallidus, external segment, right |
| TG | transverse gyri, right |
| PoG-pcs | postcentral gyrus, left, bank of the posterior central sulcus |
| PCLa-s | paracentral lobule, anterior part, right, superior bank of gyrus |
| AnG-i | angular gyrus, left, inferior bank of gyrus |
| SPL-i | superior parietal lobule, left, inferior bank of gyrus |
| HCd | head of caudate nucleus, right |
| AOrG | anterior orbital gyrus, right |
| LOrG | lateral orbital gyrus, right |
| orIFG | inferior frontal gyrus, orbital part, left |
| GRe | gyrus rectus, right |
| IRoG | inferior rostral gyrus, left |
| SRoG | superior rostral gyrus, right |
| OTG-s | occipito-temporal gyrus, left, superior bank of gyrus |
| LiG-pest | lingual gyrus, right, peristriate |
| LiG-str | lingual gyrus, right, striate |
| Cun-pest | cuneus, right, peristriate |
| SOG-s | superior occipital gyrus, left, superior bank of gyrus |
| AnG-s | angular gyrus, right, superior bank of gyrus |
| Pcu-i | precuneus, right, inferior lateral bank of gyrus |
| SPL-s | superior parietal lobule, right, superior bank of gyrus |
| Pcu-s | precuneus, right, superior lateral bank of gyrus |
| MOrG | medial orbital gyrus, left |
| Cun-str | cuneus, left, striate |
| FP-s | frontal pole, left, superior aspect |
| CA2 | CA2 field, right |
| CA3 | CA3 field, right |
| LHM | lateral hypothalamic area, mammillary region, left |
| PHA | posterior hypothalamic area, left |
| VTA | ventral tegmental area, left |
| 3 | oculomotor nuclear complex, left |
| MPB | medial parabrachial nucleus, left |
| Pr5 | principal sensory nucleus of trigeminal nerve, left |
| 7 | facial motor nucleus, left |
| 12 | hypoglossal nucleus, right |
| COMA | cortico-medial group, left |
| RaM | raphe nuclei of medulla |
| Mo5 | motor nucleus of trigeminal nerve, right |
| IO | inferior olivary complex, right |
| LPB | lateral parabrachial nucleus, left |
| LC | locus ceruleus, right |
| Pn | pontine nuclei, right |
| SubC | nucleus subceruleus, left |
| PRF | pontine reticular formation, left |
| Arc | arcuate nucleus of medulla, right |
| MBRF | midbrain reticular formation, left |
| SubCn | subcuneiform nucleus, right |
| SOC | superior olivary complex, left |
| 4 | trochlear nucleus, right |
| MBRa | midbrain raphe nuclei |
| fro | frontal operculum, right |
| opIFG | inferior frontal gyrus, opercular part, right |
| Ve-VIIAt | VIIAt |
| PV-Crus I | Crus I, left, paravermis |
| PV-VIIIA | VIIIA, right, paravermis |
| MG | medial geniculate complex, left |
| Glo | globose nucleus, left |
| DTLd | lateral group of nuclei, left, dorsal division |
| DTP | posterior group of nuclei, left |
| SC | superior colliculus, left |
| SptN | septal nuclei, left |
| PrOR | preoptic region, left |
| SO | supraoptic nucleus, left |
| PTec | pretectal region |
| Hm | medial habenular nucleus, right |
| Hl | lateral habenular nucleus, right |
| PaOG | parolfactory gyri, left |
| SCG | subcallosal cingulate gyrus, right |
| GiRt | gigantocellular group, left |
| LMRt | lateral medullary reticular group, right |
| Sp5 | spinal trigeminal nucleus, left |
| 8Ve | vestibular nuclei, left |
| 10 | dorsal motor nucleus of the vagus, left |
| 6 | abducens nucleus, left |
| RPn | pontine raphe nucleus |
| 8Co | cochlear nuclei, right |
| trIFG | inferior frontal gyrus, triangular part, left |
| POrG | posterior orbital gyrus, right |
| TP-m | temporal pole, right, medial aspect |
| TP-s | temporal pole, right, superior aspect |
| TP-i | temporal pole, right, inferior aspect |
| CGS | central glial substance |
| Cu | cuneate nucleus, left |
| CMRt | central medullary reticular group, left |
| Gr | gracile nucleus, right |
| PCLp-cs | paracentral lobule, posterior part, right, bank of cingulate sulcus |
| PCLp-l | paracentral lobule, posterior part, right, lateral bank of gyrus |
| PV-VI | VI, right, paravermis |
| Acb | nucleus accumbens, left |
| He-VI | VI, left, lateral hemisphere |
| He-Crus I | Crus I, left, lateral hemisphere |
| He-Crus II | Crus II, left, lateral hemisphere |
| He-VIIB | VIIB, left, lateral hemisphere |
| He-VIIIA | VIIIA, left, lateral hemisphere |
| Ve-I-II | I-II |
| Ve-III | III |
| Ve-IX | IX |
| Ve-IV | IV |
| Ve-V | V |
| Ve-VI | VI |
| Ve-VIIAf | VIIAf |
| Ve-VIIB | VIIB |
| Ve-VIIIA | VIIIA |
| Ve-VIIIB | VIIIB |
| PV-III | III, left, paravermis |
| PV-IV | IV, left, paravermis |
| PV-V | V, left, paravermis |
| PV-VIIIB | VIIIB, left, paravermis |
| PV-IX | IX, left, paravermis |
| PV-Crus II | Crus II, left, paravermis |
| PV-VIIB | VIIB, left, paravermis |
| FPi | frontal pole, left, inferior aspect |
| FPm | frontal pole, left, medial aspect |
| Ve-X | X |
| PV-X | X, left, paravermis |
| CgGr-s | cingulate gyrus, retrosplenial part, right, superior bank of gyrus |
| CgGr-i | cingulate gyrus, retrosplenial part, right, inferior bank of gyrus |
| PTG | paraterminal gyrus, right |
| IOG-s | inferior occipital gyrus, right, superior bank of gyrus |
| SOG-i | superior occipital gyrus, right, inferior bank of gyrus |
| IOG-i | inferior occipital gyrus, right, inferior bank of gyrus |
| CGPo | central gray of the pons, left |
| EW | Edinger-Westphal nucleus, right |
| Dk | nucleus of Darkschewitsch, left |
| ICjl | interstitial nucleus of Cajal, right |
| IC | inferior colliculus, left |
| CnF | cuneiform nucleus, right |
| ARH | arcuate nucleus of the hypothalamus, left |
| PVH | paraventricular nucleus of the hypothalamus, left |
| LHA | lateral hypothalamic area, anterior region, left |
| AHA | anterior hypothalamic area, left |
| VMH | ventromedial hypothalamic nucleus, left |
| MB | mammillary body, left |
| PCLa | paracentral lobule, anterior part, left |
| LTu | lateral tuberal nucleus, left |
| PeF | perifornical nucleus, left |
| nbM | basal nucleus of meynert, left |
| DBh | nucleus of the diagonal band, left, vertical division |
| DBv | nucleus of the diagonal band, left, horizontal division |
| CPLV | choroid plexus of the lateral ventricle |
| SuM | supramammillary nucleus, left |
| LM | lateral mammillary nucleus, left |
| MM | medial mammillary nucleus, left |
| LHT | lateral hypothalamic area, tuberal region, left |
| TM | tuberomammillary nucleus, left |
| DMH | dorsomedial hypothalamic nucleus, left |
| BST | bed nucleus of stria terminalis, left |
| OlfT | olfactory tubercle, left |
| He-IX | IX, left, lateral hemisphere |
| He-V | V, left, lateral hemisphere |
| He-IV | IV, left, lateral hemisphere |
| He-III | III, left, lateral hemisphere |
| He-VIIIB | VIIIB, left, lateral hemisphere |
| PIN | pineal gland |
| Pir | piriform cortex, left |
| PalHy | pallidohypothalamic nucleus, left |
| He-X | X, left, lateral hemisphere |
