# Supplementary material for: Brain areas affected by intranasal oxytocin show higher oxytocin receptor expression
Source: Eur J Neurosci. 2021 Sep 16;54(7):6374–81. doi: 10.1111/ejn.15447 (PMC9291869; doi:10.1111/ejn.15447)
Supplement: Supplementary file 2 — Table S2. Table of descriptive data of all 39 fMRI studies included in the meta‐analysis by Grace et al. [file EJN-54-6374-s006.docx]

| **Reference**  Details of studies included in the meta-analysis by Grace et al (*n* = 39) | **N** | **Sex** | **Age (M ±** **SD)** | **OXT dose (IU)** | **Design** | **Task** | **Reported contrasts** | **N (foci)** | | **Scanner** | **Processing Software** | **MNI or TAL** | **Statistical threshold**  ***p-value*** |
| --- | --- | --- | --- | --- | --- | --- | --- | --- | --- | --- | --- | --- | --- |
| *Emotion/Face Studies:* | | | | | | | | | | | | | |
| Domes *et al* (2007) | 13 | M | 25.7 ± 2.9 | 24 | WS | EFMT (Emotional Faces Memory Task) | PBO>OXT | 13 | | 3T Siemens | SPM2 | MNI | < 0.001 uncorrected |
| Domes *et al* (2010) | 60 | F | 24.2 ± 2.5 | 24 | WS | EFMT | OXT>PBO (fearful>neutral)  OXT>PBO (angry>neutral)  OXT>PBO (happy>neutral)  PBO>OXT (fearful>neutral)  PBO>OXT (happy>neutral) | 8  5  6  1  1 | | 1.5T Siemens | SPM5 | MNI | < 0.05, FWE corrected |
| Domes *et al* (2013) | 14 | M | 24.0 ± 6.9 | 24 | WS | Face discrimination task | PBO>OXT (faces > houses) | 1 | | 1.5T Siemens | SPM8 | MNI | < 0.05, SVC corrected |
| Domes *et al* (2014) | 14 | M | 24.0 ± 6.0 | 24 | WS | Face emotion recognition (section of eyes or mouth) | OXT> PBO (eyes)  OXT>PBO (mouth) | 1  1 | | 1.5 T Siemens | SPM8 | MNI | < 0.001, uncorrected |
| Eckstein *et al* (2015) | 62 | M | 24.61 ± 4.28 | 24 | BS | Fear conditioning | OXT>PBO (CS+>CS-) | 3 | | 1.5T Siemens | SPM8 | MNI | *p*< 0.05, FWE corrected |
| Eckstein *et al* (2016) | 97 | M | 24.45 ± 4.02 | 24 | BS | Fear conditioning | PBO>OXT (shock>baseline) | 2 | | 1.5T Siemens | SPM8 | MNI | < 0.05, FWE corrected |
| Grimm *et al* (2014) | 32 | M | 28.4 ± 4.5 | 24 | WS | Stress task | OXT>PBO | 4 | | 3T Siemens | SPM8 | MNI | *<* 0.05, FWE corrected |
| Kanat *et al* (2015b) | 49 | M | 24.11 ± 3.01 | 24 | BS | Masked face emotions (attend to eyes or mouth) | OXT<PBO (angry; eyes)  OXT>PBO (angry; mouth)  OXT<PBO (angry: eye>mouth)  OXT<PBO (happy; mouth)  OXT<PBO (happy; mouth>eyes) | 14  1  13  2  6 | | 3T Siemens | SPM | MNI | < 0.001, uncorrected |
| Labuschagne *et al* (2012) | 18 | M | 29.4 ± 9.0 | 24 | WS | EFMT | PBO>OXT (sad>neutral)  OXT>PBO (sad>neutral)  PBO>OXT (happy>neutral)  OXT>PBO (happy>neutral) | 4  1  7  1 | | 3T Siemens | SPM5 | MNI | *<* 0.005, uncorrected |
| Lischke *et al* (2012) | 14 | F | 23.79 ± 2.32 | 24 | WS | Threatening scenes | OXT>PBO (negative-neutral)  PBO>OXT (positive-neutral) | 4  1 | | 1.5T Siemens | SPM8 | MNI | *<* 0.001, uncorrected |
| Petrovic *et al* (2008) | 30 | M | 24.85 | 32 | BS | Fear conditioning of faces | PBO>OXT (fear conditioning)  PBO>OXT (direct gaze faces)  OXT>PBO (direct gaze faces)  PBO>OXT (averted gaze faces)  OXT>PBO (averted gaze faces)  PBO>OXT (direct>averted gaze) | 5  1  5  2  3  4 | | 1.5T Siemens | SPM5 | MNI | *<* 0.001, uncorrected |
| Pincus *et al* (2010) | 9 | M/F | 35.5 ± 10.62 | 40 | WS | RMET | OXT>PBO | 18 | | 3T Phillips | FSL | MNI | *<* 0.05, cluster corrected |
| Scheele *et al* (2014c) | 23 | M | 25.75 ± 3.82 | 24 | WS | Emotional face matching | OXT>PBO (disgust>neutral) | 2 | | 3T Siemens | SPM8 | MNI | *<* 0.005, FWE corrected |
| Striepens *et al* (2012) | 70 | M | 25.35 ± 4.37 | 24 | BS | Aversive social stimuli | OXT>PBO (remembered>not remembered) | 1 | | 1.5T Siemens | SPM8 | MNI | *<* 0.05, FWE corrected |
| Zunhammer *et al* (2015) | 30 | M | 24.9 | 40 | WS | Thermal pain | OXT>PBO | 8 | | 3T Siemens | SPM8 | MNI | *<* 0.001, uncorrected |
| *Studies of social processes:* | | | | | | | | | | | | | |
| Baumgartner *et al* (2008) | 49 | M | 21.7 ± 2.5 | 24 | BS | Trust feedback | OXT>PBO (risk game, prefeedback)  PBO>OXT (trust game, postfeedback) | | 2  2 | 3T Phillips | SPM5 | MNI | *<* 0.005, uncorrected |
| Bos *et al* (2015) | 24 | M | 23.1 | 24 | WS | Pain (observed) | PBO<OXT | | 5 | 3T Phillips | SPM8 | MNI | *<* 0.05, FWE corrected |
| Chen *et al* (2017) | 57 | M/F | M: 20.9 ± 1.6  F: 20.5 ± 1.4 | 24 | WS | Trust | PBO>OXT (women) | | 1 | 3T  Siemens | FSL | MNI | *<* 0.05, FWE corrected |
| Cohen *et al* (2017) | 19 | M | 26.05 ± 3.51 | 24 | WS | Prisoners Dilemma | OXT>PBO (friend>stranger) | | 6 | 3T GE | BrainVoyager | TAL | *<* 0.05, FDR corrected |
| Eckstein *et al* (2014) | 60 | M | 24.67 ± 3.89 | 24 | BS | Psychosocial stress | OXT>PBO (stress > no stress) | | 3 | 1.5T Siemens | SPM8 | MNI | *<* 0.05, FWE corrected |
| Feng *et al* (2015a) | 186 | M/F | 20.7 | 24 | BS | Prisoner’s Dilemma | OXT-PBO male > OXT-PBO female | | 8 | 3T Siemens | FSL | MNI | *<* 0.05, FWE corrected |
| Gozzi *et al* (2017) | 21 | M | 26.57 | 24 | WS | Aversive (negative social feedback) | PBO>OXT | | 10 | 3T GE | SPM8 | MNI | *<* 0.001, uncorrected |
| Groppe *et al* (2013) | 28 | F | 26.64 ± 5.55 | 26 | BS | Social reward vs. punishment | OXT>PBO (reward anticipation)  OXT<PBO (punishment anticipation)  OXT>PBO (punishment+reward) | | 3  4  8 | 3T Siemens | SPM8 | MNI | *<* 0.001, uncorrected |
| Gao *et al* (2016) | 74 | M/F | 22.8 ± 1.7 | 24 | BS | First-Impression Task | OXT-PBO male > OXT-PBO female | | 1 | 3T Siemens | SPM8 | MNI | *<* 0.05, FWE corrected |
| Hecht *et al* (2017) | 28 | F | 23.08 ± 0.73 | 24 | WS | Social Videos | OXT<PBO (women) | | 2 | 3T Siemens | FSL | MNI | *<* 0.05, FWE corrected |
| Hu *et al* (2015) | 54 | M | 19.8 ± 1.49 | 24 | BS | Social feedback | OXT>PBO  OXT>PBO (learning: non-social light)  OXT>PBO (learning: social emoticon)  OXT>PBO (learning: social female)  OXT>PBO (feedback: non-social light)  OXT>PBO feedback: social emoticon)  OXT>PBO (feedback: social female) | | 3  8  4  10  3  2  3 | 3T Siemens | SPM8 | MNI | *<* 0.05, FDR corrected |
| Hu *et al* (2016) | 22 | M | 25.1 ± 3.88 | 24 | WS | Motivation (monetary/altruism) | OXT>PBO (prosocial decision) | | 3 | 3T Siemens | SPM8 | MNI | *<* 0.05, FWE corrected; |
| Li *et al* (2017) | 15 | M | 32.8 ± 4.7 | 24 | WS | Parental caregiving | OXT>PBO | | 3 | 3T Siemens | FSL | MNI | *<* 0.005, uncorrected |
| Mickey *et al* (2016) | 20 | M | 22.0 ± 2 | 24 | WS | Motivation (money) | PBO>OXT | | 7 | 3T Philips | SPM | MNI | *<* 0.001, uncorrected |
| Preckel *et al* (2015) - Experiment 1 | 48 | M | 24.6 ± 4.56 | 24 | BS | Moral reasoning | PBO>OXT (moral>non-moral) | | 4 | 1.5T Siemens | SPM8 | MNI | *<* 0.05, FWE corrected |
| Riem *et al* (2012) | 42 | F | 29.07 ± 7.56 | 24 | BS | Responses to infant crying | OXT>PBO (cry>control) | | 2 | 3T Siemens | FSL | MNI | *<* 0.05, cluster corrected |
| Rilling *et al* (2012) | 91 | M | 20.2 | 40 | BS | Prisoner’s dilemma | OXT>PBO (human-computer)  OXT>PBO (unreciprocated cooperation)  PBO>OXT (unreciprocated cooperation)  OXT>PBO (cooperation)  OXT>PBO (partner defection)  PBO>OXT (partner defection) | | 2  1  1  10  1  1 | 3T Siemens | Brain voyager | TAL | *<* 0.05, corrected for multiple comparisons |
| Rilling *et al* (2014) | 121 (63/58) | M/F | F: 20.4 | 24 | BS | Prisoner’s Dilemma | OXT<PBO (M+F; human - computer)  OXT>PBO (M-F; human - computer)  OXT>PBO (M>F; choice human- choice computer) | | 4  11  10 | 3T Siemens | Brain Voyager | TAL | *<* 0.05, corrected for multiple comparisons |
| Scheele *et al* (2014a) | 40 | M | 25.75 ± 3.82 | 24 | WS | Emotional ratings of touch | OXT>PBO (female touch)  OXT>PBO (female>male) | | 4  2 | 3T Siemens | SPM8 | MNI | *<* 0.05, FWE corrected |
| Scheele *et al* (2016) | 40 | F | 24.38 ± 3.26 | 24 | WS | Approach (pair bonding) | OXT>PBO (FWE corrected) | | 4 | 3T Siemens | SPM8 | MNI | *<* 0.05, FWE corrected |
| Singer *et al* (2008) | 20 | M | 24.6 ± 3.2 | 32 | WS | Empathy for pain | OXT>PBO (self)  PBO>OXT (self)  PBO>OXT (other)  PBO>OXT (prosocial>selfish)  PBO>OXT (selfish>prosocial) | | 4  2  1  3  2 | 1.5T Siemens | SPM5 | MNI | *<* 0.05, FWE corrected |
| Striepens *et al* (2016) | 31 | F | 25.35 ± 4.37 | 24 | WS | Motivation (food) | OXT>PBO (later)  OXT>PBO (now)  OXT>PBO (now>later)  OXT>PBO (later>now) | | 10  3  3  8 | 3T  Siemens | FSL | MNI | *<* 0.05, FWE corrected |
| Wittfoth-Schardt *et al* (2012) | 21 | M | 39.3 ± 6.2 | 24 | WS | Fathers viewing children | OXT<PBO (own>familiar child)  OXT>PBO (own>unfamiliar child)  OXT>PBO (unfamiliar>familiar child) | | 1  1  7 | 1.5T Siemens | SPM8 | MNI | *<* 0.001, uncorrected |
| Zhao *et al* (2016) | 41 | M | 22.83 ± 0.34 | 24 | BS | Social (altruism/selfish) | OXT<PBO | | 17 | 3T GE | SPM | MNI | *<* 0.01, FDR corrected |
