# Supplementary figures and images for: Brain areas affected by intranasal oxytocin show higher oxytocin receptor expression
Source: Eur J Neurosci. 2021 Sep 16;54(7):6374–81. doi: 10.1111/ejn.15447 (PMC9291869; doi:10.1111/ejn.15447)

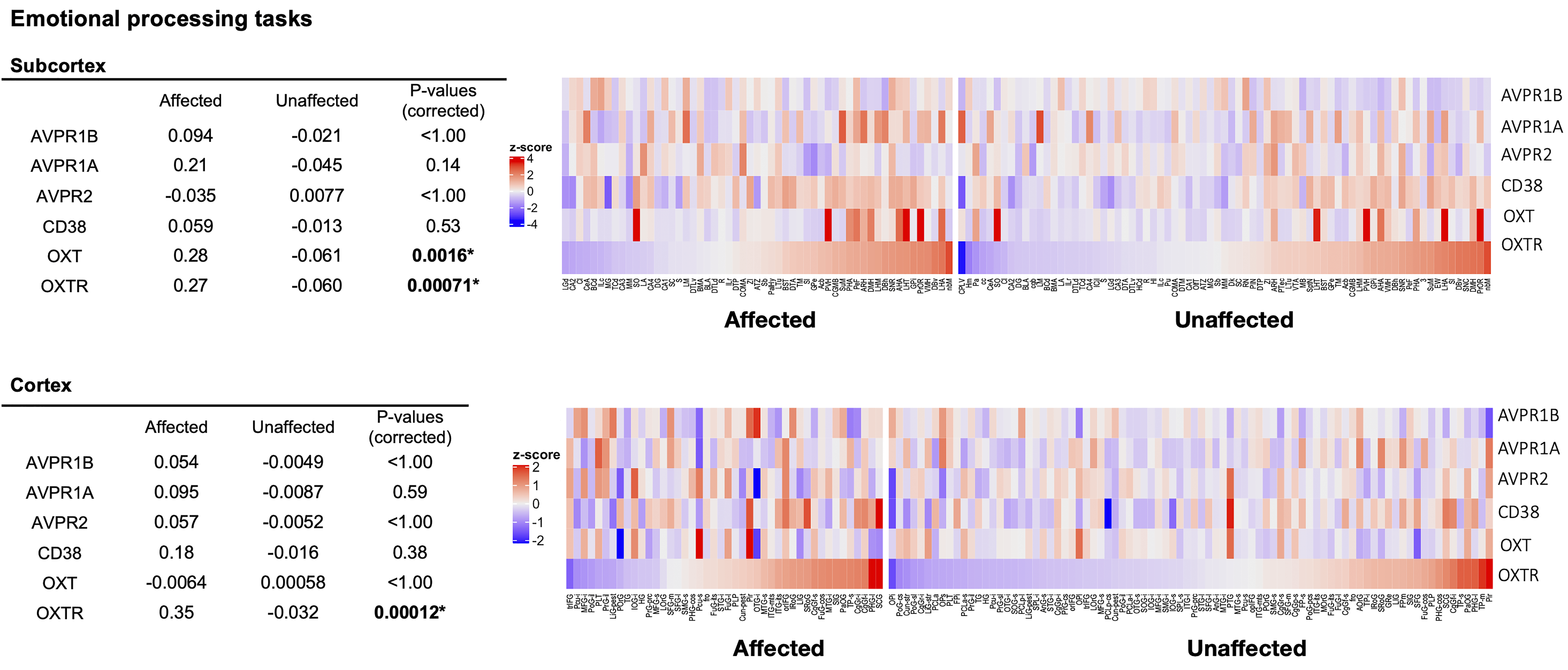

Supplement: Supplementary file 3 — Figure S1. Emotional processing results for all genes. Differences in average z‐normalized expression values for all genes of interest in affected versus unaffected subcortical and cortical samples using the emotional processing p‐statistic map. Heatmaps show average expression of genes per brain structure. Brain structure abbreviations are adopted from the AHBA data. [file EJN-54-6374-s005.tif]

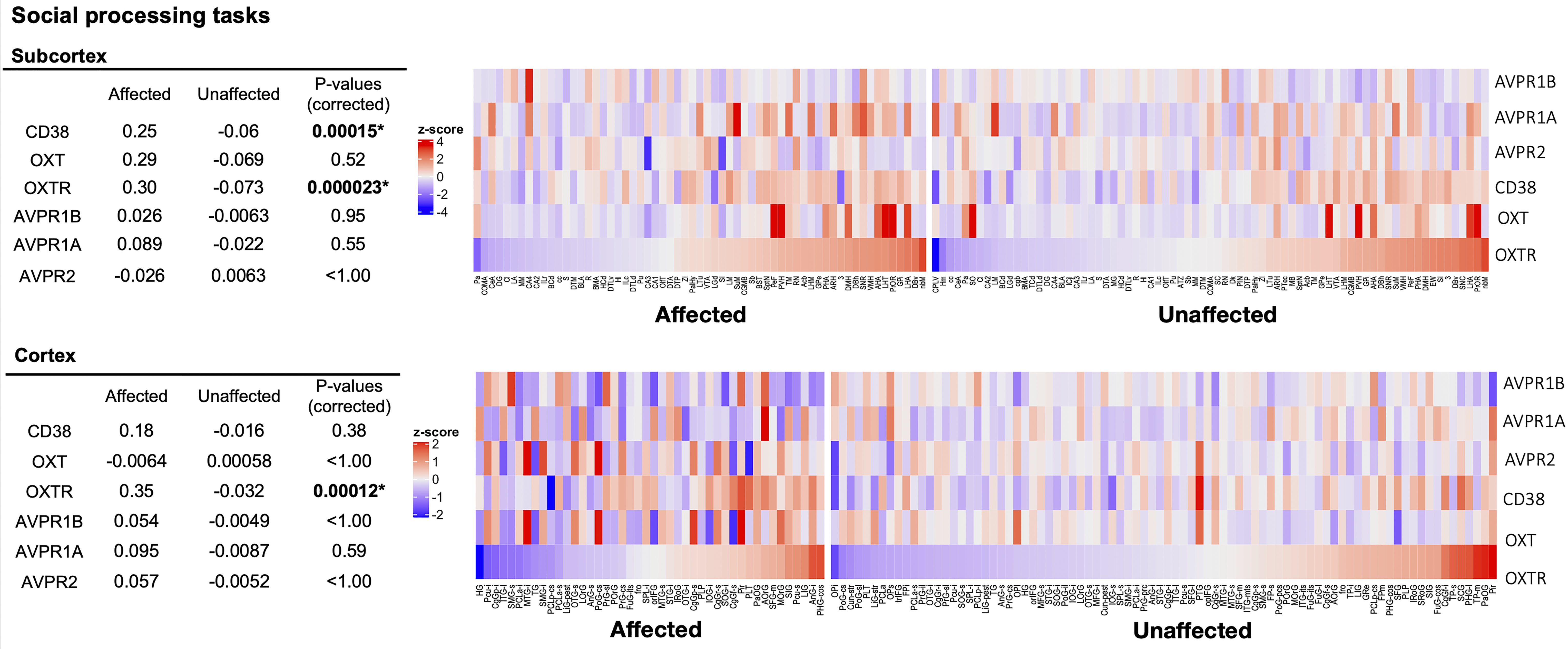

Supplement: Supplementary file 4 — Figure S2. Social processing results for all genes. Similar to S1 Fig, but results are shown for the social processing mask. [file EJN-54-6374-s004.tif]

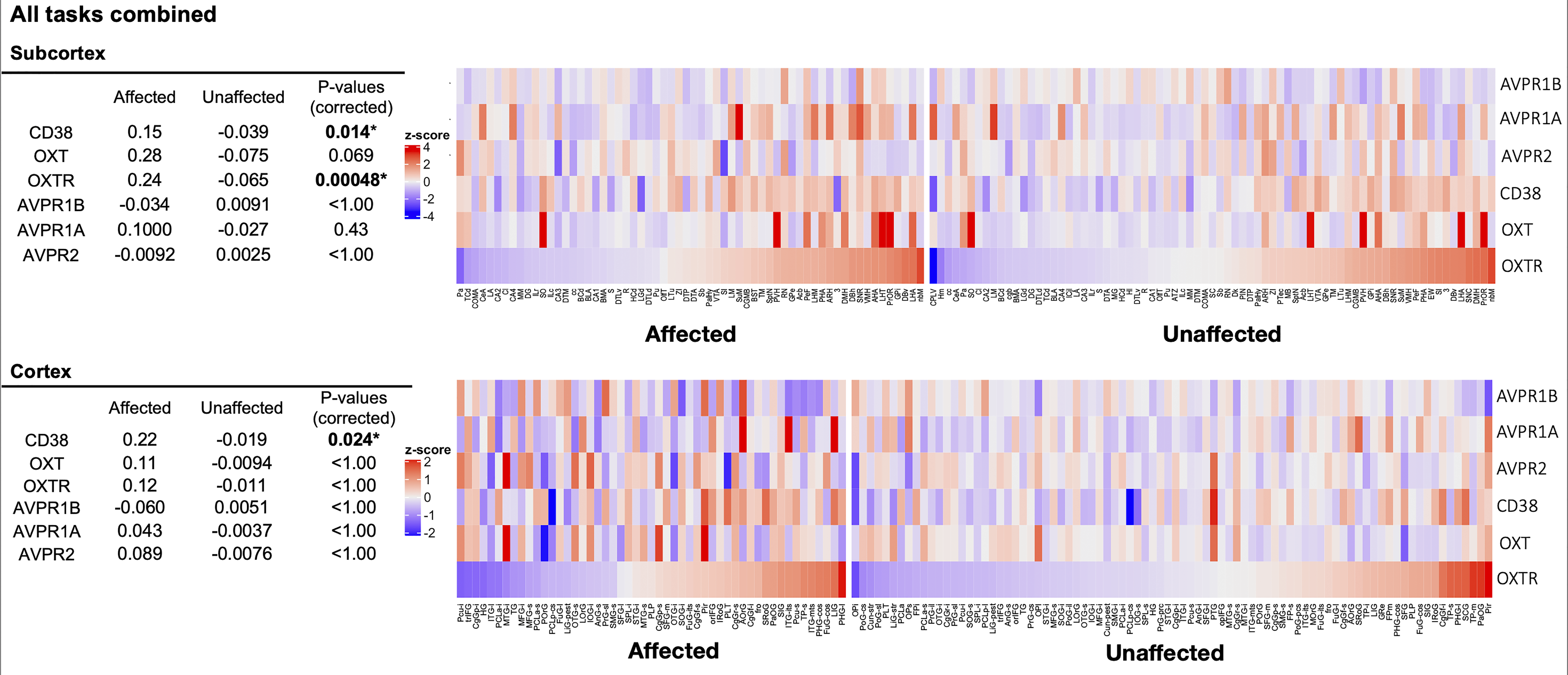

Supplement: Supplementary file 5 — Figure S3. All tasks results for all genes. Similar to S1 Fig, but results are shown for the all‐tasks map. [file EJN-54-6374-s001.tif]

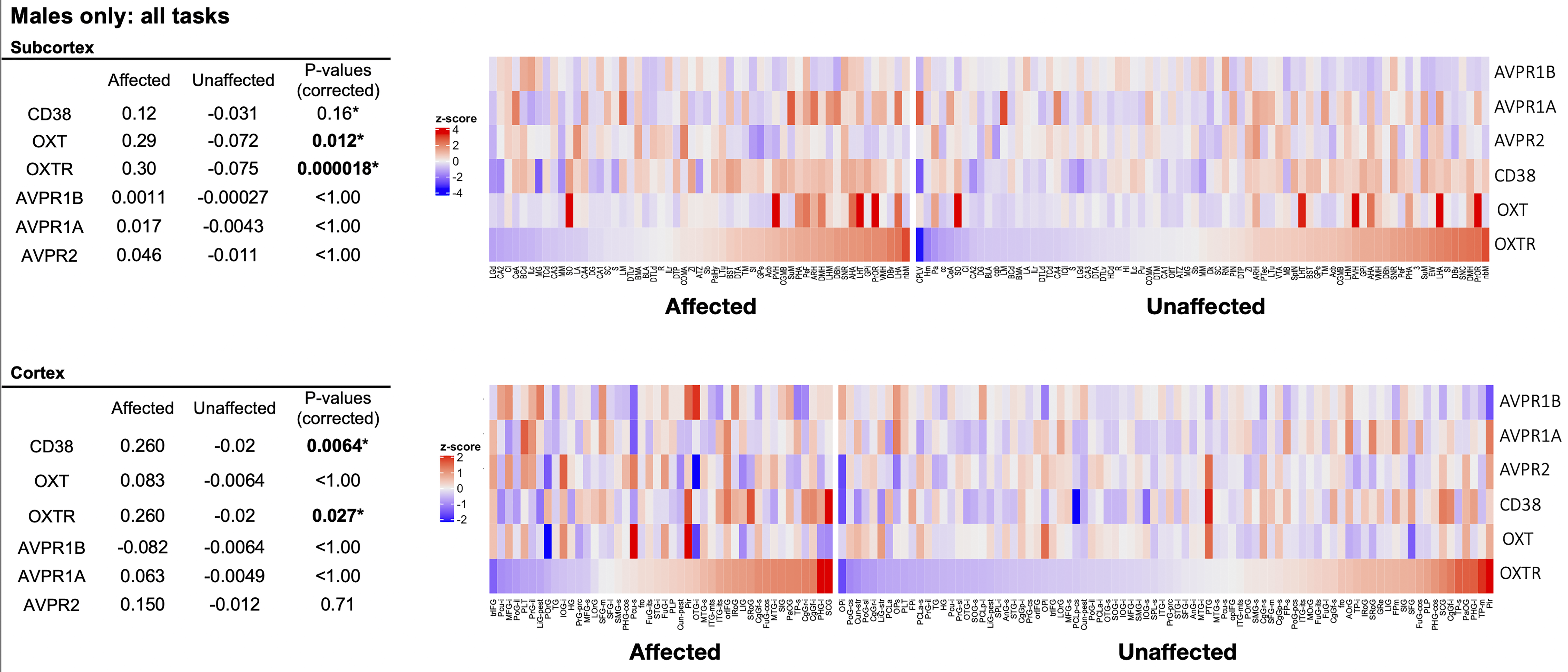

Supplement: Supplementary file 6 — Figure S4. Male‐only results for all genes. Similar to S1 Fig, but results are shown for the male‐only all‐tasks map, with only five male donor brains included in the analysis. [file EJN-54-6374-s007.tif]
