# Supplementary material for: Brain areas affected by intranasal oxytocin show higher oxytocin receptor expression
Source: Eur J Neurosci. 2021 Sep 16;54(7):6374–81. doi: 10.1111/ejn.15447 (PMC9291869; doi:10.1111/ejn.15447)

Supplemental Data

S1 - Structure acronyms (as they appear in Figure 2) and their corresponding names, extracted from the AHBA sample information for all six donor brains.

Stucture Acronym	Structure Name
PCLa-i	paracentral lobule, anterior part, right, inferior bank of gyrus
Cl	claustrum, right
LGd	dorsal lateral geniculate nucleus, left
CA4	CA4 field, right
DG	dentate gyrus, right
Dt	dentate nucleus, left
Fas	fastigial nucleus, left
Emb	emboliform nucleus, right
S	subiculum, left
CA1	CA1 field, left
TCd	tail of caudate nucleus, left
DTA	anterior group of nuclei, right
DTLv	lateral group of nuclei, right, ventral division
ILr	rostral group of intralaminar nuclei, right
DTM	medial group of nuclei, left
ILc	caudal group of intralaminar nuclei, left
R	reticular nucleus of thalamus, right
Pa	paraventricular nuclei, right of thalamus, right
SI	substantia innominata, right
Sb	subthalamic nucleus, right
SNC	substantia nigra, pars compacta, left
ZI	zona incerta, left
CGMB	central gray substance of midbrain, right
RN	red nucleus, left
SNR	substantia nigra, pars reticulata, left
MTG-i	middle temporal gyrus, left, inferior bank of gyrus
ATZ	amygdalohippocampal transition zone, left
BLA	basolateral nucleus, left
BMA	basomedial nucleus, left
LA	lateral nucleus, left
CeA	central nucleus, left
SIG	short insular gyri, left
PoG-cs	postcentral gyrus, right, bank of the central sulcus
OTG-i	occipito-temporal gyrus, left, inferior bank of gyrus
FuG-its	fusiform gyrus, left, bank of the its
FuG-l	fusiform gyrus, left, lateral bank of gyrus
FuG-cos	fusiform gyrus, left, bank of cos
HG	Heschl's gyrus, left
Pu	putamen, left
LIG	long insular gyri, right
STG-i	superior temporal gyrus, left, inferior bank of gyrus
MTG-s	middle temporal gyrus, left, superior bank of gyrus
ITG-l	inferior temporal gyrus, left, lateral bank of gyrus
ITG-mts	inferior temporal gyrus, left, bank of mts
STG-l	superior temporal gyrus, right, lateral bank of gyrus
ITG-its	inferior temporal gyrus, right, bank of the its
PoG-il	postcentral gyrus, right, inferior lateral aspect of gyrus
PLT	planum temporale, right
PrG-prc	precentral gyrus, left, bank of the precentral sulcus
PrG-sl	precentral gyrus, left, superior lateral aspect of gyrus

PrG-il	precentral gyrus, left, inferior lateral aspect of gyrus
MFG-i	middle frontal gyrus, left, inferior bank of gyrus
SFG-m	superior frontal gyrus, left, medial bank of gyrus
SFG-l	superior frontal gyrus, left, lateral bank of gyrus
MFG-s	middle frontal gyrus, left, superior bank of gyrus
PrG-cs	precentral gyrus, right, bank of the central sulcus
GPi	globus pallidus, internal segment, right
PHG-l	parahippocampal gyrus, left, lateral bank of gyrus
PHG-cos	parahippocampal gyrus, left, bank of the cos
CgGp-s	cingulate gyrus, parietal part, left, superior bank of gyrus
CgGp-i	cingulate gyrus, parietal part, left, inferior bank of gyrus
cc	corpus callosum
cgb	cingulum bundle, right
PLP	planum polare, left
SMG-s	supramarginal gyrus, left, superior bank of gyrus
BCd	body of caudate nucleus, right
SMG-i	supramarginal gyrus, left, inferior bank of gyrus
CgGf-s	cingulate gyrus, frontal part, left, superior bank of gyrus
CgGf-i	cingulate gyrus, frontal part, left, inferior bank of gyrus
PoG-sl	postcentral gyrus, right, superior lateral aspect of gyrus
GPe	globus pallidus, external segment, right
TG	transverse gyri, right
PoG-pcs	postcentral gyrus, left, bank of the posterior central sulcus
PCLa-s	paracentral lobule, anterior part, right, superior bank of gyrus
AnG-i	angular gyrus, left, inferior bank of gyrus
SPL-i	superior parietal lobule, left, inferior bank of gyrus
HCd	head of caudate nucleus, right
AOrG	anterior orbital gyrus, right
LOrG	lateral orbital gyrus, right
orIFG	inferior frontal gyrus, orbital part, left
GRe	gyrus rectus, right
IRoG	inferior rostral gyrus, left
SRoG	superior rostral gyrus, right
OTG-s	occipito-temporal gyrus, left, superior bank of gyrus
LiG-pest	lingual gyrus, right, peristriate
LiG-str	lingual gyrus, right, striate
Cun-pest	cuneus, right, peristriate
SOG-s	superior occipital gyrus, left, superior bank of gyrus
AnG-s	angular gyrus, right, superior bank of gyrus
Pcu-i	precuneus, right, inferior lateral bank of gyrus
SPL-s	superior parietal lobule, right, superior bank of gyrus
Pcu-s	precuneus, right, superior lateral bank of gyrus
MOrG	medial orbital gyrus, left
Cun-str	cuneus, left, striate
FP-s	frontal pole, left, superior aspect
CA2	CA2 field, right
CA3	CA3 field, right
LHM	lateral hypothalamic area, mammillary region, left
PHA	posterior hypothalamic area, left
VTA	ventral tegmental area, left
3	oculomotor nuclear complex, left
MPB	medial parabrachial nucleus, left
Pr5	principal sensory nucleus of trigeminal nerve, left
7	facial motor nucleus, left
12	hypoglossal nucleus, right
COMA	cortico-medial group, left
RaM	raphe nuclei of medulla
Mo5	motor nucleus of trigeminal nerve, right
IO	inferior olivary complex, right
LPB	lateral parabrachial nucleus, left

LC	locus ceruleus, right
Pn	pontine nuclei, right
SubC	nucleus subceruleus, left
PRF	pontine reticular formation, left
Arc	arcuate nucleus of medulla, right
MBRF	midbrain reticular formation, left
SubCn	subcuneiform nucleus, right
SOC	superior olivary complex, left
4	trochlear nucleus, right
MBRa	midbrain raphe nuclei
fro	frontal operculum, right
opIFG	inferior frontal gyrus, opercular part, right
Ve-VIIAt	VIIAt
PV-Crus I	Crus I, left, paravermis
PV-VIIIA	VIIIA, right, paravermis
MG	medial geniculate complex, left
Glo	globose nucleus, left
DTLd	lateral group of nuclei, left, dorsal division
DTP	posterior group of nuclei, left
SC	superior colliculus, left
SptN	septal nuclei, left
PrOR	preoptic region, left
SO	supraoptic nucleus, left
PTec	pretectal region
Hm	medial habenular nucleus, right
Hl	lateral habenular nucleus, right
PaOG	parolfactory gyri, left
SCG	subcallosal cingulate gyrus, right
GiRt	gigantocellular group, left
LMRt	lateral medullary reticular group, right
Sp5	spinal trigeminal nucleus, left
8Ve	vestibular nuclei, left
10	dorsal motor nucleus of the vagus, left
6	abducens nucleus, left
RPn	pontine raphe nucleus
8Co	cochlear nuclei, right
trIFG	inferior frontal gyrus, triangular part, left
POrG	posterior orbital gyrus, right
TP-m	temporal pole, right, medial aspect
TP-s	temporal pole, right, superior aspect
TP-i	temporal pole, right, inferior aspect
CGS	central glial substance
Cu	cuneate nucleus, left
CMRt	central medullary reticular group, left
Gr	gracile nucleus, right
PCLp-cs	paracentral lobule, posterior part, right, bank of cingulate sulcus
PCLp-l	paracentral lobule, posterior part, right, lateral bank of gyrus
PV-VI	VI, right, paravermis
Acb	nucleus accumbens, left
He-VI	VI, left, lateral hemisphere
He-Crus I	Crus I, left, lateral hemisphere
He-Crus II	Crus II, left, lateral hemisphere
He-VIIB	VIIB, left, lateral hemisphere
He-VIIIA	VIIIA, left, lateral hemisphere
Ve-I-II	I-II
Ve-III	III
Ve-IX	IX
Ve-IV	IV
Ve-V	V

Ve-VI	VI
Ve-VIIAf	VIIAf
Ve-VIIB	VIIB
Ve-VIIIA	VIIIA
Ve-VIIIB	VIIIB
PV-III	III, left, paravermis
PV-IV	IV, left, paravermis
PV-V	V, left, paravermis
PV-VIIIB	VIIIB, left, paravermis
PV-IX	IX, left, paravermis
PV-Crus II	Crus II, left, paravermis
PV-VIIB	VIIB, left, paravermis
FPi	frontal pole, left, inferior aspect
FPm	frontal pole, left, medial aspect
Ve-X	X
PV-X	X, left, paravermis
CgGr-s	cingulate gyrus, retrosplenial part, right, superior bank of gyrus
CgGr-i	cingulate gyrus, retrosplenial part, right, inferior bank of gyrus
PTG	paraterminal gyrus, right
IOG-s	inferior occipital gyrus, right, superior bank of gyrus
SOG-i	superior occipital gyrus, right, inferior bank of gyrus
IOG-i	inferior occipital gyrus, right, inferior bank of gyrus
CGPo	central gray of the pons, left
EW	Edinger-Westphal nucleus, right
Dk	nucleus of Darkschewitsch, left
ICjl	interstitial nucleus of Cajal, right
IC	inferior colliculus, left
CnF	cuneiform nucleus, right
ARH	arcuate nucleus of the hypothalamus, left
PVH	paraventricular nucleus of the hypothalamus, left
LHA	lateral hypothalamic area, anterior region, left
AHA	anterior hypothalamic area, left
VMH	ventromedial hypothalamic nucleus, left
MB	mammillary body, left
PCLa	paracentral lobule, anterior part, left
LTu	lateral tuberal nucleus, left
PeF	perifornical nucleus, left
nbM	basal nucleus of meynert, left
DBh	nucleus of the diagonal band, left, vertical division
DBv	nucleus of the diagonal band, left, horizontal division
CPLV	choroid plexus of the lateral ventricle
SuM	supramammillary nucleus, left
LM	lateral mammillary nucleus, left
MM	medial mammillary nucleus, left
LHT	lateral hypothalamic area, tuberal region, left
TM	tuberomammillary nucleus, left
DMH	dorsomedial hypothalamic nucleus, left
BST	bed nucleus of stria terminalis, left
OlfT	olfactory tubercle, left
He-IX	IX, left, lateral hemisphere
He-V	V, left, lateral hemisphere
He-IV	IV, left, lateral hemisphere
He-III	III, left, lateral hemisphere
He-VIIIB	VIIIB, left, lateral hemisphere
PIN	pineal gland
Pir	piriform cortex, left
PalHy	pallidohypothalamic nucleus, left
He-X	X, left, lateral hemisphere

S2 - Details of studies included in the meta-analysis by Grace et al (*n* = 39)

**Reference**	**N**	**Sex**	**Age (M ± SD)**	**OXT dose (IU)**	**Design**	**Task**	**Reported contrasts**	**N (foci)**	**Scanner**	**Processing Software**	**MNI or TAL**	**Statistical threshold *p-value***
Emotion/Face Studies:												
Domes *et al* (2007)	13	M	25.7 ± 2.9	24	WS	EFMT (Emotional Faces Memory Task)	PBO>OXT	13	3T Siemens	SPM2	MNI	< 0.001 uncorrected
Domes *et al* (2010)	60	F	24.2 ± 2.5	24	WS	EFMT	OXT>PBO (fearful>neutral)<br>OXT>PBO (angry>neutral)<br>OXT>PBO (happy>neutral)<br>PBO>OXT (fearful>neutral)<br>PBO>OXT (happy>neutral)	8<br>5<br>6<br>1<br>1	1.5T Siemens	SPM5	MNI	< 0.05, FWE corrected
Domes *et al* (2013)	14	M	24.0 ± 6.9	24	WS	Face discrimination task	PBO>OXT (faces > houses)	1	1.5T Siemens	SPM8	MNI	< 0.05, SVC corrected
Domes *et al* (2014)	14	M	24.0 ± 6.0	24	WS	Face emotion recognition (section of eyes or mouth)	OXT> PBO (eyes)<br>OXT>PBO (mouth)	1<br>1	1.5 T Siemens	SPM8	MNI	< 0.001, uncorrected
Eckstein *et al* (2015)	62	M	24.61 ± 4.28	24	BS	Fear conditioning	OXT>PBO (CS+>CS-)	3	1.5T Siemens	SPM8	MNI	*p*< 0.05, FWE corrected
Eckstein *et al* (2016)	97	M	24.45 ± 4.02	24	BS	Fear conditioning	PBO>OXT (shock>baseline)	2	1.5T Siemens	SPM8	MNI	< 0.05, FWE corrected
Grimm *et al* (2014)	32	M	28.4 ± 4.5	24	WS	Stress task	OXT>PBO	4	3T Siemens	SPM8	MNI	< 0.05, FWE corrected
Kanat *et al* (2015b)	49	M	24.11 ± 3.01	24	BS	Masked face emotions (attend to eyes or mouth)	OXT<PBO (angry; eyes)<br>OXT>PBO (angry; mouth)<br>OXT<PBO (angry: eye>mouth)<br>OXT<PBO (happy; mouth)<br>OXT<PBO (happy; mouth>eyes)	14<br>1<br>13<br>2<br>6	3T Siemens	SPM	MNI	< 0.001, uncorrected

Labuschagne *et al* (2012)	18	M	29.4 ± 9.0	24	WS	EFMT	PBO>OXT (sad>neutral)<br>OXT>PBO (sad>neutral)<br>PBO>OXT (happy>neutral)<br>OXT>PBO (happy>neutral)	4<br>1<br>7<br>1	3T Siemens	SPM5	MNI	< 0.005, uncorrected
Lischke *et al* (2012)	14	F	23.79 ± 2.32	24	WS	Threatening scenes	OXT>PBO (negative-neutral)<br>PBO>OXT (positive-neutral)	4<br>1	1.5T Siemens	SPM8	MNI	< 0.001, uncorrected
Petrovic *et al* (2008)	30	M	24.85	32	BS	Fear conditioning of faces	PBO>OXT (fear conditioning)<br>PBO>OXT (direct gaze faces)<br>OXT>PBO (direct gaze faces)<br>PBO>OXT (averted gaze faces)<br>OXT>PBO (averted gaze faces)<br>PBO>OXT (direct>averted gaze)	5<br>1<br>5<br>2<br>3<br>4	1.5T Siemens	SPM5	MNI	< 0.001, uncorrected
Pincus *et al* (2010)	9	M/F	35.5 ± 10.62	40	WS	RMET	OXT>PBO	18	3T Phillips	FSL	MNI	< 0.05, cluster corrected
Scheele *et al* (2014c)	23	M	25.75 ± 3.82	24	WS	Emotional face matching	OXT>PBO (disgust>neutral)	2	3T Siemens	SPM8	MNI	< 0.005, FWE corrected
Striepens *et al* (2012)	70	M	25.35 ± 4.37	24	BS	Aversive social stimuli	OXT>PBO (remembered>not remembered)	1	1.5T Siemens	SPM8	MNI	< 0.05, FWE corrected
Zunhammer *et al* (2015)	30	M	24.9	40	WS	Thermal pain	OXT>PBO	8	3T Siemens	SPM8	MNI	< 0.001, uncorrected
Studies of social processes:												
Baumgartner *et al* (2008)	49	M	21.7 ± 2.5	24	BS	Trust feedback	OXT>PBO (risk game, prefeedback)	2<br>2	3T Phillips	SPM5	MNI	< 0.005, uncorrected

							PBO>OXT (trust game, postfeedback)				
Bos *et al* (2015)	24	M	23.1	24	WS	Pain (observed)	PBO<OXT	5	3T Phillips	SPM8	MNI	< 0.05, FWE corrected
Chen *et al* (2017)	57	M/F	M: 20.9 ± 1.6<br>F: 20.5 ± 1.4	24	WS	Trust	PBO>OXT (women)	1	3T Siemens	FSL	MNI	< 0.05, FWE corrected
Cohen *et al* (2017)	19	M	26.05 ± 3.51	24	WS	Prisoners Dilemma	OXT>PBO (friend>stranger)	6	3T GE	BrainVoyager	TAL	< 0.05, FDR corrected
Eckstein *et al* (2014)	60	M	24.67 ± 3.89	24	BS	Psychosocial stress	OXT>PBO (stress > no stress)	3	1.5T Siemens	SPM8	MNI	< 0.05, FWE corrected
Feng *et al* (2015a)	186	M/F	20.7	24	BS	Prisoner's Dilemma	OXT-PBO male > OXT-PBO female	8	3T Siemens	FSL	MNI	< 0.05, FWE corrected
Gozzi *et al* (2017)	21	M	26.57	24	WS	Aversive (negative social feedback)	PBO>OXT	10	3T GE	SPM8	MNI	< 0.001, uncorrected
Groppe *et al* (2013)	28	F	26.64 ± 5.55	26	BS	Social reward vs. punishment	OXT>PBO (reward anticipation)<br>OXT<PBO (punishment anticipation)<br>OXT>PBO (punishment+reward)	3<br>4<br>8	3T Siemens	SPM8	MNI	< 0.001, uncorrected
Gao *et al* (2016)	74	M/F	22.8 ± 1.7	24	BS	First-Impression Task	OXT-PBO male > OXT-PBO female	1	3T Siemens	SPM8	MNI	< 0.05, FWE corrected
Hecht *et al* (2017)	28	F	23.08 ± 0.73	24	WS	Social Videos	OXT<PBO (women)	2	3T Siemens	FSL	MNI	< 0.05, FWE corrected
Hu *et al* (2015)	54	M	19.8 ± 1.49	24	BS	Social feedback	OXT>PBO<br>OXT>PBO (learning: non-social light)<br>OXT>PBO (learning: social emoticon)<br>OXT>PBO (learning: social female)<br>OXT>PBO (feedback: non-social light)	3<br>8<br>4<br>10<br>3<br>2<br>3	3T Siemens	SPM8	MNI	< 0.05, FDR corrected

							OXT>PBO feedback: social emoticon)<br>OXT>PBO (feedback: social female)					
Hu *et al* (2016)	22	M	25.1 ± 3.88	24	WS	Motivation (monetary/altruism)	OXT>PBO (prosocial decision)	3	3T Siemens	SPM8	MNI	< 0.05, FWE corrected;
Li *et al* (2017)	15	M	32.8 ± 4.7	24	WS	Parental caregiving	OXT>PBO	3	3T Siemens	FSL	MNI	< 0.005, uncorrected
Mickey *et al* (2016)	20	M	22.0 ± 2	24	WS	Motivation (money)	PBO>OXT	7	3T Philips	SPM	MNI	< 0.001, uncorrected
Preckel *et al* (2015) - Experiment 1	48	M	24.6 ± 4.56	24	BS	Moral reasoning	PBO>OXT (moral>non-moral)	4	1.5T Siemens	SPM8	MNI	< 0.05, FWE corrected
Riem *et al* (2012)	42	F	29.07 ± 7.56	24	BS	Responses to infant crying	OXT>PBO (cry>control)	2	3T Siemens	FSL	MNI	< 0.05, cluster corrected
Rilling *et al* (2012)	91	M	20.2	40	BS	Prisoner's dilemma	OXT>PBO (human-computer)<br>OXT>PBO (unreciprocated cooperation)<br>PBO>OXT (unreciprocated cooperation)<br>OXT>PBO (cooperation)<br>OXT>PBO (partner defection)<br>PBO>OXT (partner defection)	2<br>1<br>1<br>10<br>1<br>1	3T Siemens	Brain voyager	TAL	< 0.05, corrected for multiple comparisons
Rilling *et al* (2014)	121 (63/58)	M/F	F: 20.4	24	BS	Prisoner's Dilemma	OXT<PBO (M+F; human - computer)<br>OXT>PBO (M-F; human - computer)<br>OXT>PBO (M>F; choice human-choice computer)	4<br>11<br>10	3T Siemens	Brain Voyager	TAL	< 0.05, corrected for multiple comparisons
Scheele *et al* (2014a)	40	M	25.75 ± 3.82	24	WS	Emotional ratings of touch	OXT>PBO (female touch)<br>OXT>PBO (female>male)	4<br>2	3T Siemens	SPM8	MNI	< 0.05, FWE corrected

Scheele *et al* (2016)	40	F	24.38 ± 3.26	24	WS	Approach (pair bonding)	OXT>PBO (FWE corrected)	4	3T Siemens	SPM8	MNI	< 0.05, FWE corrected
Singer *et al* (2008)	20	M	24.6 ± 3.2	32	WS	Empathy for pain	OXT>PBO (self)<br>PBO>OXT (self)<br>PBO>OXT (other)<br>PBO>OXT (prosocial>selfish)<br>PBO>OXT (selfish>prosocial)	4<br>2<br>1<br>3<br>2	1.5T Siemens	SPM5	MNI	< 0.05, FWE corrected
Striepens *et al* (2016)	31	F	25.35 ± 4.37	24	WS	Motivation (food)	OXT>PBO (later)<br>OXT>PBO (now)<br>OXT>PBO (now>later)<br>OXT>PBO (later>now)	10<br>3<br>3<br>8	3T Siemens	FSL	MNI	< 0.05, FWE corrected
Wittfoth-Schardt *et al* (2012)	21	M	39.3 ± 6.2	24	WS	Fathers viewing children	OXT<PBO (own>familiar child)<br>OXT>PBO (own>unfamiliar child)<br>OXT>PBO (unfamiliar>familiar child)	1<br>1<br>7	1.5T Siemens	SPM8	MNI	< 0.001, uncorrected
Zhao *et al* (2016)	41	M	22.83 ± 0.34	24	BS	Social (altruism/selfish)	OXT<PBO	17	3T GE	SPM	MNI	< 0.01, FDR corrected

Emotional processing tasks - Figure S1

Subcortex

	Affected	Unaffected	P-values (corrected)
AVPR1B	0.094	-0.021	<1.00
AVPR1A	0.21	-0.045	0.14
AVPR2	-0.035	0.0077	<1.00
CD38	0.059	-0.013	0.53
OXT	0.28	-0.061	**0.0016***
OXTR	0.27	-0.060	**0.00071***

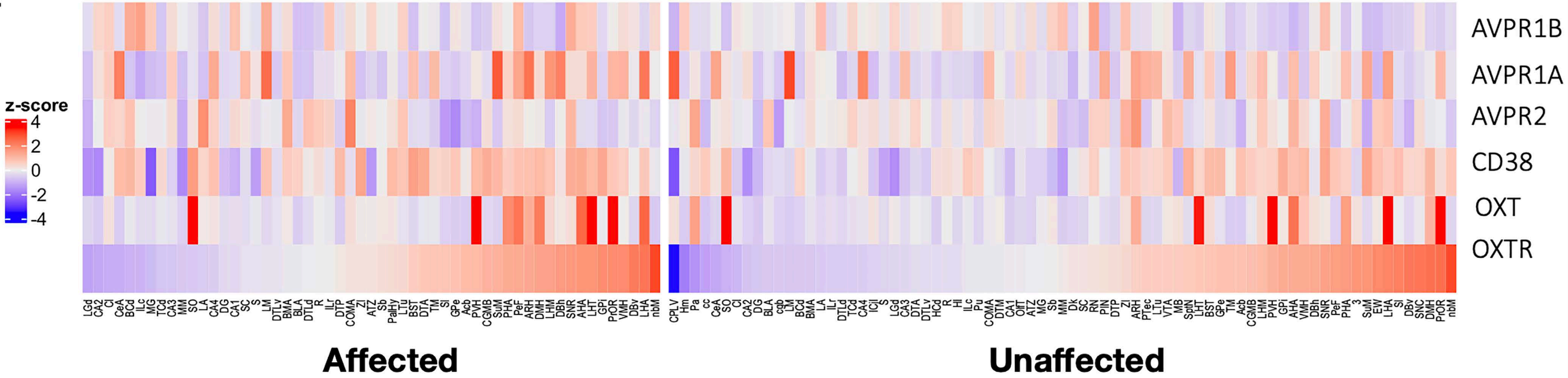

Cortex

	Affected	Unaffected	P-values (corrected)
AVPR1B	0.054	-0.0049	<1.00
AVPR1A	0.095	-0.0087	0.59
AVPR2	0.057	-0.0052	<1.00
CD38	0.18	-0.016	0.38
OXT	-0.0064	0.00058	<1.00
OXTR	0.35	-0.032	**0.00012***

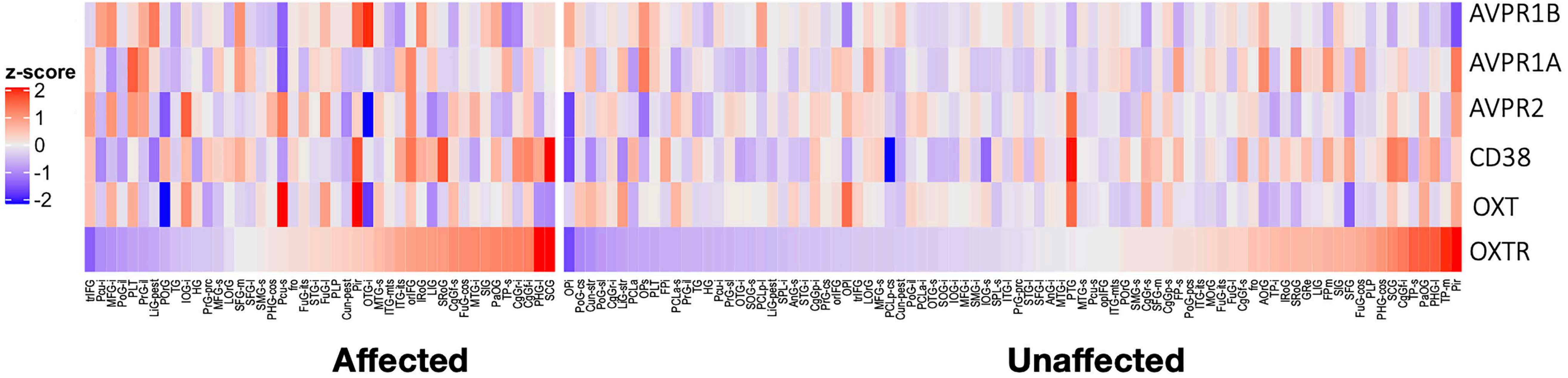

Social processing tasks - Figure S2

Subcortex

	Affected	Unaffected	P-values (corrected)
CD38	0.25	-0.06	**0.00015***
OXT	0.29	-0.069	0.52
OXTR	0.30	-0.073	**0.000023***
AVPR1B	0.026	-0.0063	0.95
AVPR1A	0.089	-0.022	0.55
AVPR2	-0.026	0.0063	<1.00

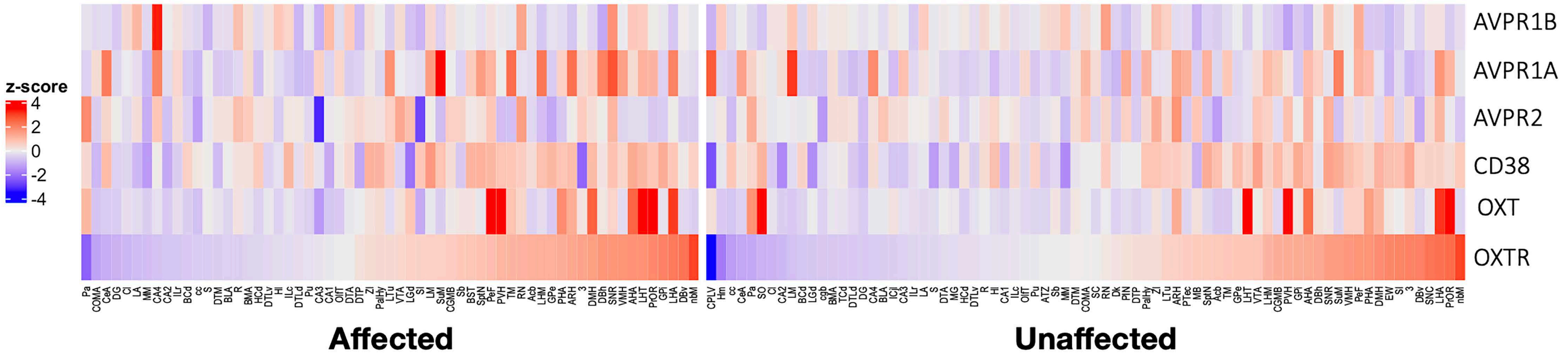

Cortex

	Affected	Unaffected	P-values (corrected)
CD38	0.18	-0.016	0.38
OXT	-0.0064	0.00058	<1.00
OXTR	0.35	-0.032	**0.00012***
AVPR1B	0.054	-0.0049	<1.00
AVPR1A	0.095	-0.0087	0.59
AVPR2	0.057	-0.0052	<1.00

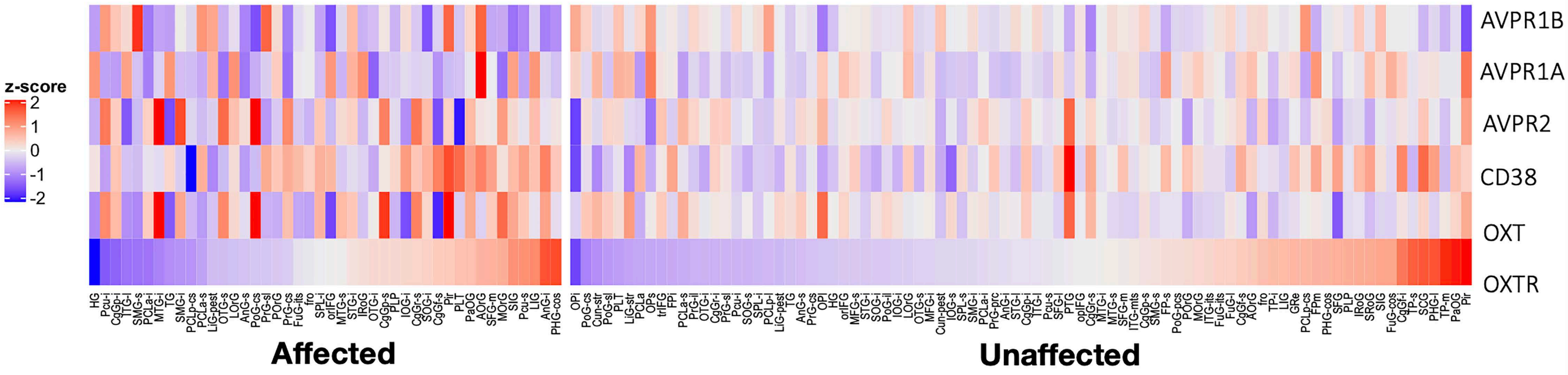

All tasks combined - Figure S3

Subcortex

	Affected	Unaffected	P-values (corrected)
CD38	0.15	-0.039	**0.014***
OXT	0.28	-0.075	0.069
OXTR	0.24	-0.065	**0.00048***
AVPR1B	-0.034	0.0091	<1.00
AVPR1A	0.1000	-0.027	0.43
AVPR2	-0.0092	0.0025	<1.00

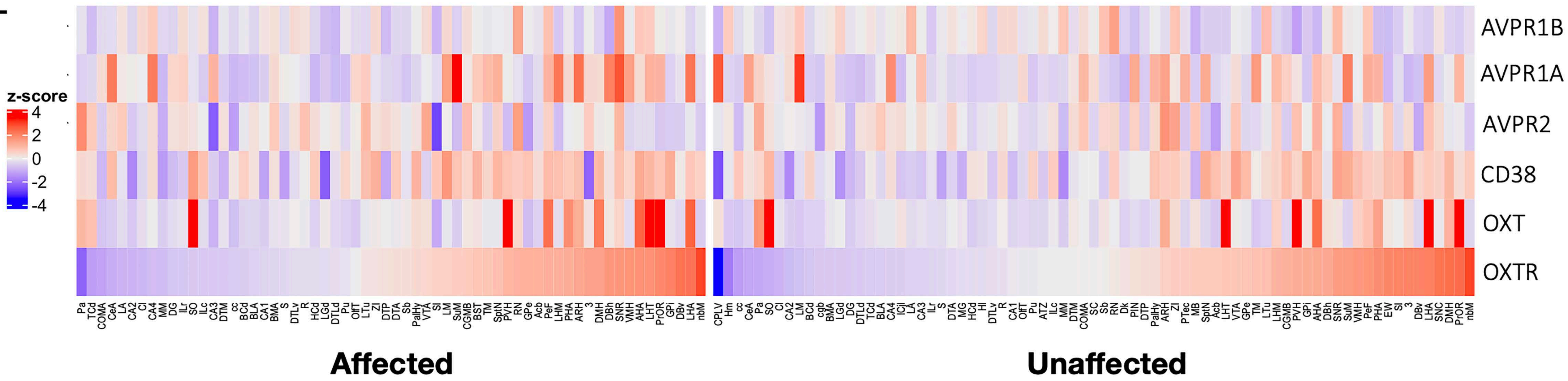

Cortex

	Affected	Unaffected	P-values (corrected)
CD38	0.22	-0.019	**0.024***
OXT	0.11	-0.0094	<1.00
OXTR	0.12	-0.011	<1.00
AVPR1B	-0.060	0.0051	<1.00
AVPR1A	0.043	-0.0037	<1.00
AVPR2	0.089	-0.0076	<1.00

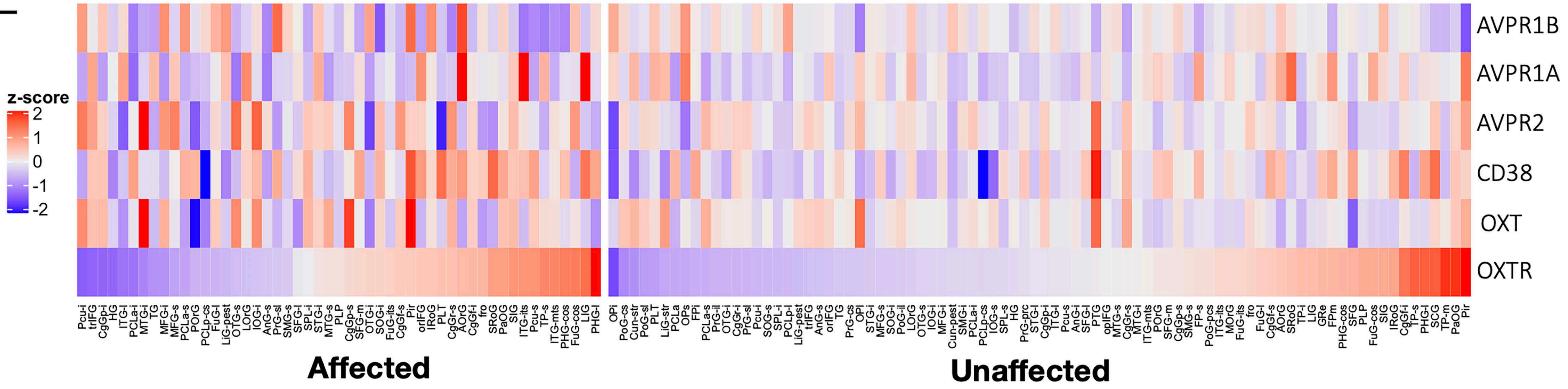

Males only: all tasks - Figure S4

Subcortex

	Affected	Unaffected	P-values (corrected)
CD38	0.12	-0.031	0.16*
OXT	0.29	-0.072	**0.012***
OXTR	0.30	-0.075	**0.000018***
AVPR1B	0.0011	-0.00027	<1.00
AVPR1A	0.017	-0.0043	<1.00
AVPR2	0.046	-0.011	<1.00

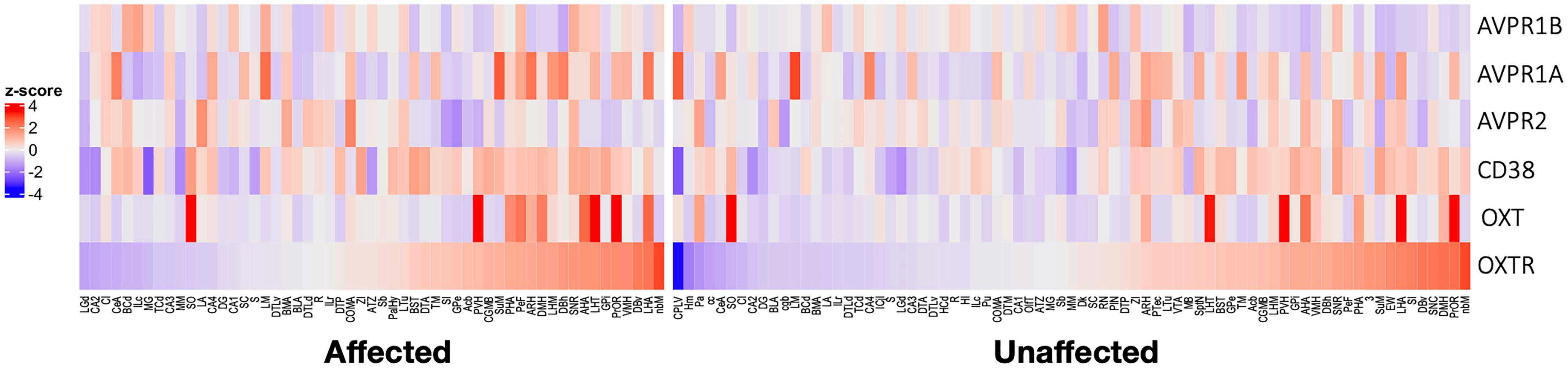

Cortex

	Affected	Unaffected	P-values (corrected)
CD38	0.260	-0.02	**0.0064***
OXT	0.083	-0.0064	<1.00
OXTR	0.260	-0.02	**0.027***
AVPR1B	-0.082	-0.0064	<1.00
AVPR1A	0.063	-0.0049	<1.00
AVPR2	0.150	-0.012	0.71

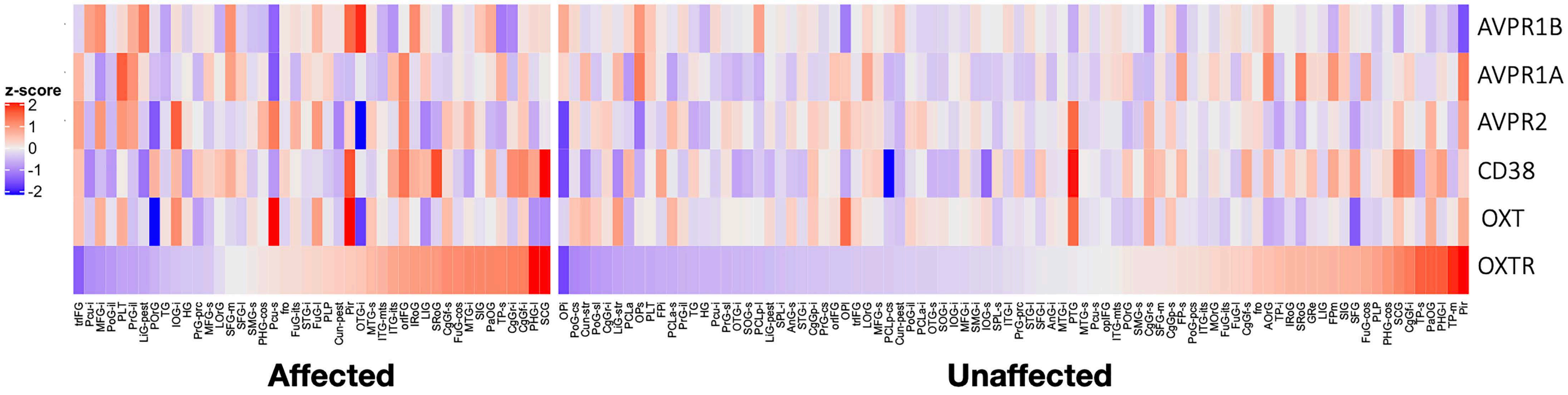

Supplement: Supplementary file 7 — Data S1. Supplementary Files. [file EJN-54-6374-s002.pdf]
